# Benefit-risk profiling of advanced therapies in moderate-to-severe ulcerative colitis: a clustered network meta-analysis

**DOI:** 10.3389/fphar.2026.1961820

**Published:** 2026-10-14

**Authors:** Xinyuan Zhang, Hao Qian, Lijuan Yang, Hao Wang, Guangjun Sun, Aizhen Lin

**Affiliations:** 1 Hubei University of Chinese Medicine, Wuhan, China; 2 Shanghai University of Traditional Chinese Medicine, Shanghai, China; 3 Department of Anorectal Medicine, Hubei Provincial Hospital of Traditional Chinese Medicine, Affiliated Hospital of Hubei University of Chinese Medicine, Hubei Province Academy of Traditional Chinese Medicine, Wuhan, China; 4 Hubei Shizhen Laboratory, Wuhan, China; 5 Hubei Key Laboratory of theory and application research of liver and kidney in traditional Chinese medicine, Wuhan, China

**Keywords:** bivariate clustered ranking analysis, IL-23 antagonists, network meta-analysis, ulcerative colitis, upadacitinib

## Abstract

**Background and Aims:**

The therapeutic landscape for moderate-to-severe ulcerative colitis (UC) has expanded with the introduction of oral small molecules and recently approved biologics. However, the scarcity of head-to-head trials necessitates indirect comparisons to clarify their relative positioning. We aimed to determine the comparative efficacy and tolerability hierarchy of approved and emerging advanced therapies, incorporating the most contemporary evidence.

**Methods:**

We retrieved randomized controlled trials (RCTs) from major databases up to November 2025. Efficacy outcomes included clinical remission and endoscopic improvement; tolerability was evaluated via discontinuations due to adverse events. We employed Bayesian network meta-analysis and bivariate clustered ranking to visualize efficacy-tolerability profiles, stratifying by prior biologic exposure.

**Results:**

A total of 32 RCTs were included. In the induction phase, Upadacitinib, Ustekinumab, and Risankizumab co-occupied the “most favorable” quadrant, combining high efficacy with superior tolerability. In the maintenance phase, Upadacitinib 30 mg demonstrated the highest-ranking probability for efficacy, while IL-23 antagonists (Guselkumab, Mirikizumab) and low-dose Upadacitinib exhibited a highly favorable efficacy-tolerability profile. Notably, in biologic-exposed patients, Upadacitinib demonstrated the highest relative efficacy among evaluated therapies, whereas Guselkumab emerged as a highly effective biologic alternative based on current estimates.

**Conclusion:**

Based on current network estimates, Upadacitinib is suggested among the most efficacious options for achieving clinical and endoscopic endpoints, particularly in refractory disease, while IL-23 antagonists present highly favorable tolerability profiles for maintenance without compromising efficacy. These findings support a tailored approach to drug selection, aligning patient-specific profiles of inflammatory burden with current evidence-based safety thresholds.

**Systematic Review Registration:**

https://www.crd.york.ac.uk/PROSPERO/view/CRD420261292029, identifier CRD420261292029.

## 1 Introduction

Ulcerative colitis (UC) is a chronic immune-mediated disease characterized by diffuse inflammation of the colonic mucosa. It most commonly begins in adolescence or early adulthood but can develop at almost any age, with a peak in young adulthood (late teens–30 s) and a smaller second peak later in life, typically following a relapsing–remitting course (Damianos et al., 2025). The long-term disease activity significantly impairs patients’ physical and mental health, leading to reduced quality of life and work capacity; some patients also develop psychiatric symptoms such as depression. Furthermore, with prolonged disease duration, the risk of colorectal cancer rises substantially, and there is no curative treatment for UC (Selvakumar and Samsudin, 2025).

Over the past decade, the management of moderate-to-severe active UC has undergone a major shift. Treatment is no longer limited to conventional corticosteroids and thiopurines, but has rapidly expanded to include various advanced targeted therapies with distinct mechanisms of action. These agents include tumor necrosis factor (TNF) antagonists, gut-selective anti-integrins (e.g., vedolizumab), interleukin-12/23 (IL-12/23) inhibitors (e.g., ustekinumab), and selective interleukin-23 (IL-23) inhibitors (Singh et al., 2024). In addition, the introduction of oral small-molecule drugs such as Janus kinase (JAK) inhibitors and sphingosine-1-phosphate (S1P) receptor modulators has further improved treatment convenience and efficacy. These advances have shifted the treatment goal from mere symptom control to achieving deep remission—marked by mucosal healing—thereby improving long-term disease outcomes (Shehab et al., 2025).

However, the growing array of therapies also poses challenges for clinical decision-making. Although authoritative recommendations such as the 2024 American College of Gastroenterology clinical practice guidelines are available (Singh et al., 2024), the precise positioning of different drugs remains unclear due to a lack of direct comparative evidence. Aside from the notable exception of the VARSITY trial, which directly compared vedolizumab and adalimumab (Sands et al., 2019a), head-to-head randomized controlled trials (RCTs) remain scarce. Consequently, clinicians often must rely on indirect evidence when making decisions, particularly when weighing the potent efficacy of small-molecule drugs against the favorable safety profile of newer biologics (Magro et al., 2025).

Network meta-analysis (NMA) has become essential for bridging evidence gaps through the synthesis of direct and indirect comparisons (Rouse et al., 2017). However, existing NMAs often assess efficacy and safety separately, overlooking the balance required for informed clinical decision-making. Additionally, recent landmark trials, including the QUASAR study and long-term Upadacitinib extensions, have not been integrated into previous syntheses, potentially rendering their findings outdated. To address these limitations, this study employs a Bayesian network meta-analysis framework to provide a contemporary synthesis of all advanced therapies currently approved by the FDA/EMA or in late-stage clinical development for UC. Beyond standard ranking metrics, we utilize a bivariate clustered ranking approach, simultaneously mapping primary efficacy outcomes (clinical remission and endoscopic improvement) against tolerability (defined as discontinuation due to adverse events). By visualizing the two-dimensional efficacy-tolerability profile of each agent, this study aims to clarify the comparative hierarchy of advanced therapies, thereby providing a quantitative evidence base to optimize therapeutic positioning in the era of precision medicine.

## 2 Methods

### 2.1 Protocol and retrieval strategy

This systematic review and network meta-analysis (NMA) was conducted in strict accordance with the PRISMA (Preferred Reporting Items for Systematic Reviews and Meta-Analyses) extension statement for network meta-analyses (Hutton et al., 2015). The protocol was prospectively registered on PROSPERO (ID: CRD420261292029). We systematically searched PubMed, Embase and the Cochrane Library from inception to 14 November 2025. The search aimed to identify RCTs evaluating advanced therapies for moderate-to-severely active UC, using relevant Medical Subject Headings (MeSH) and free-text terms such as biologic agents, small molecules, and JAK inhibitors. The detailed search syntax is provided in Supplementary Table S1.

### 2.2 Study selection criteria

Based on the PICOS framework, the following inclusion criteria were applied: Participants: adult patients (≥18 years) diagnosed with moderate-to-severely active UC according to conventional clinical and endoscopic criteria (e.g., Mayo score). Interventions: biological agents (anti-TNF, anti-integrin, anti-IL-12/23, or anti-IL-23) or small-molecule drugs (JAK inhibitors, S1P receptor modulators) approved by the FDA or EMA for induction or maintenance therapy. Comparators: placebo or another active agent (head-to-head trials). Outcomes: Quantitative reporting of efficacy (clinical remission, endoscopic improvement) and safety endpoints (adverse events, serious adverse events). Study design: phase IIb or phase III randomized controlled trials. Exclusion criteria were: (1) observational cohort studies, single-arm studies, or *post hoc* analyses; (2) studies restricted to acute severe UC requiring hospitalization; (3) studies only assessing pharmacokinetic equivalence of biosimilars without reporting clinical efficacy data; (4) non-English publications or abstracts with insufficient data for extraction.

### 2.3 Data extraction and quality assessment

Two reviewers independently screened studies and extracted data using a standardized form. Disagreements were resolved through consensus or by consulting a third senior reviewer. Outcomes were defined as follows: induction outcomes were assessed at the trial-defined primary endpoint (weeks 6–14), and maintenance outcomes were evaluated at the end of the treatment phase (weeks 40–60) among responders to induction therapy. In alignment with the STRIDE-II consensus for treat-to-target strategies in ulcerative colitis, we selected clinical remission and endoscopic improvement as our primary efficacy outcomes, as they represent the ultimate therapeutic goals. Clinical response was not evaluated as it is generally considered an intermediate target. Furthermore, endoscopic improvement (defined as a Mayo endoscopic subscore of 0 or 1) was chosen over strict endoscopic remission (subscore of 0) because it is the most consistently reported endoscopic endpoint across the pivotal trials included in our network.

For safety and tolerability assessments, we extracted data on any adverse events (AEs), serious adverse events (SAEs), and discontinuation due to adverse events. While any AEs and SAEs were evaluated as secondary safety metrics, discontinuation due to adverse events was designated *a priori* as the primary endpoint for overall treatment tolerability. The evaluation of any AEs is notoriously confounded by high background noise. SAEs are rare and sporadically reported—often reflecting disease flares rather than genuine drug toxicity—which can introduce zero-event cells, wide credible intervals, and unstable network estimates. In contrast, discontinuation due to adverse events represents a highly pragmatic, patient-centric measure. The use of treatment discontinuation as a reliable surrogate for overall acceptability is supported by consistent methodological practice across multiple landmark network meta-analyses (Cipriani et al., 2018).

Data processing adhered to the following principles:

Dose standardization: Data corresponding to standard therapeutic doses approved by regulatory authorities (FDA/EMA) were prioritized; exploratory or unapproved doses were excluded to maintain clinical relevance. For drugs with distinct approved dosing regimens (e.g., Upadacitinib 15 and 30 mg), each dose was treated as an independent node to analyze dose–response relationships.

Route of administration pooling: Given the comparable efficacy and pharmacokinetics between intravenous and subcutaneous formulations of vedolizumab, as well as their interchangeability in clinical practice, data from both administration routes were pooled into a single “vedolizumab” node to enhance statistical power.

Data imputation: In the HIBISCUS I and II trials (Rubin et al., 2022), only aggregated efficacy data were reported for the adalimumab arm. Assuming consistency between the two trials, we imputed trial-specific event counts based on the reported overall remission rate (24%), weighted by the respective sample sizes (*n* = 142 and *n* = 143). The potential influence of this imputation approach on the robustness of the results was evaluated through sensitivity analysis, and its limitations are discussed in the study.

### 2.4 Statistical analysis

We performed Bayesian network meta-analysis using the GEMTC package (version 4.4.3) in R. For all dichotomous efficacy and tolerability outcomes, summary effect estimates were expressed as odds ratios (ORs) alongside their corresponding 95% credible intervals (CrIs). Statistical significance was established when the 95% CrI did not contain the value of 1.

Network model: A random-effects model was employed to account for potential between-study heterogeneity. Markov chain Monte Carlo (MCMC) simulation was run for 20,000 iterations after a 20,000-iteration burn-in period. Convergence was assessed using Brooks–Gelman–Rubin diagnostic plots.

Heterogeneity and consistency: Global heterogeneity for all outcomes was quantified using the posterior distribution of the between-study variance ( τ ^2^). Global inconsistency was strictly evaluated by comparing the Deviance Information Criterion (DIC) between the consistency model and the Unrelated Mean Effects (UME) model, with detailed assessment results provided in Supplementary Table S2. The transitivity assumption was evaluated qualitatively, supported by statistical summaries, by comparing the distributions of potential effect modifiers (e.g., prior biologic exposure, baseline Mayo score) across treatment comparisons. Local inconsistency between direct and indirect evidence was examined using the node-splitting method.

Publication bias: To explore the potential presence of small-study effects and publication bias within the network, we pre-specified the use of comparison-adjusted funnel plots. Active interventions were ordered by their chronological year of approval to establish a meaningful reference for the funnel plot.

Ranking metrics: Treatment ranking was assessed using the surface under the cumulative ranking curve (SUCRA). SUCRA values range from 0% to 100%, with higher values indicating a greater probability of being among the better treatments. To account for uncertainty, SUCRA rankings were interpreted alongside effect estimates and their 95% CrIs, rather than in isolation.

Bivariate clustered ranking analysis: To simultaneously evaluate efficacy and tolerability, therapies were grouped using the k-means clustering algorithm based on their SUCRA values. Prior to clustering, the SUCRA values for both axes were standardized (z-score transformed) to ensure equal weighting and to eliminate the influence of scale differences. The optimal number of clusters ( k ) was determined objectively using the silhouette width method. To assess cluster stability and confirm the robustness of the grouping, a sensitivity analysis was conducted by comparing the k-means results with hierarchical clustering algorithms, which yielded consistent therapeutic groupings.

Sensitivity and subgroup analyses: To evaluate network robustness, we conducted a targeted sensitivity analysis by integrating the HIBISCUS I and II trials into a single pooled node using their original, un-imputed adalimumab data. Furthermore, subgroup analyses were pre-specified based on key clinical effect modifiers, notably prior biologic exposure (biologic-naive vs. biologic-experienced patients), to explore potential sources of heterogeneity and validate the consistency of treatment effects across different patient populations.

## 3 Result

### 3.1 Research selection

The systematic literature search identified an initial 10,820 records. Following deduplication and step-by-step screening against the inclusion criteria, 25 reports representing 32 unique randomized controlled trials (RCTs) that met the inclusion criteria were finally included for quantitative synthesis (Sands et al., 2019a; Rubin et al., 2022; Motoya et al., 2019; Feagan et al., 2013; Sands et al., 2019b; Danese et al., 2022a; Sandborn et al., 2017; Sandborn et al., 2014a; Sandborn et al., 2014b; Louis et al., 2024; Sandborn et al., 2021; D’Haens et al., 2023; Jiang et al., 2015; Rutgeerts et al., 2005; Rubin et al., 2025; Naganuma et al., 2025; Feagan et al., 2021; Danese et al., 2022b; Vermeire et al., 2022; Sandborn et al., 2023; Sandborn et al., 2020; Hibi et al., 2017; Suzuki et al., 2014; Sandborn et al., 2012; Reinisch et al., 2011). The detailed screening process and reasons for exclusion are delineated in the PRISMA flow diagram (Figure 1).

**FIGURE 1:**
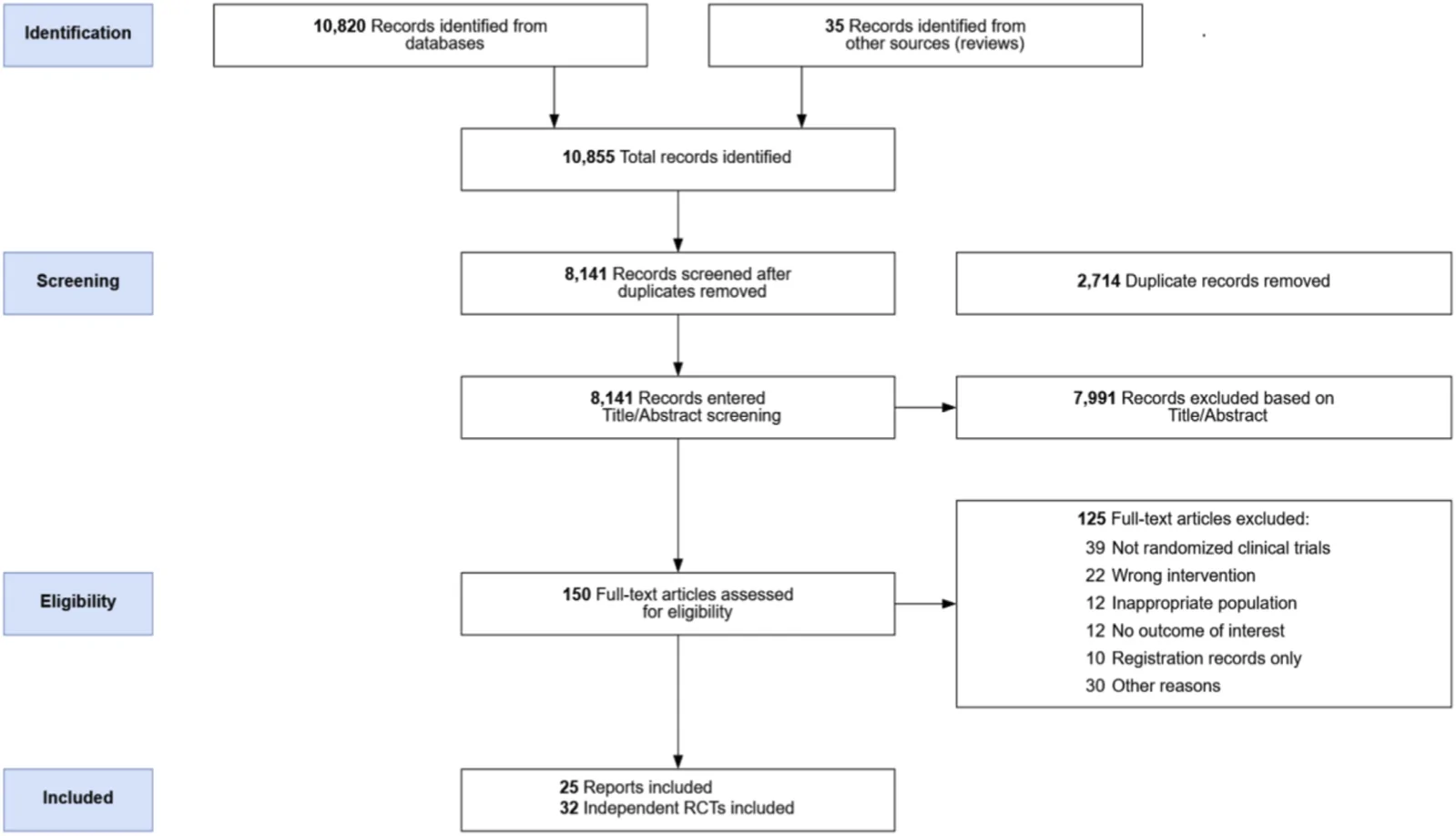
PRISMA flow diagram of the study selection process. Abbreviation: RCT, randomized controlled trial.

### 3.2 Characteristics of included studies

This network meta-analysis included a total of 14,107 adult patients with moderate-to-severe ulcerative colitis. Among the included trials, 26 were conducted in the induction phase and 20 in the maintenance phase. The evaluated interventions spanned five distinct mechanisms of action: TNF- α inhibitors, integrin antagonists, IL-12/23 or IL-23 p19 antagonists, JAK inhibitors, and S1P receptor modulators. The majority of studies were multi-center, randomized, double-blind trials. Notably, our analysis was substantially reinforced by the inclusion of five active-comparator trials from four articles, which provided crucial direct comparative evidence to the network. Detailed trial designs, dosage regimens, and patient baseline characteristics are summarized in Table 1.

**TABLE 1:** Baseline characteristics of included studies.

Study	Coverage	Number of people (F/M)	Age (years)	Mayo score	Treatment	Biologic-experienced (%)	Prior anti-TNF failure (%)^a^
Rutgeerts2005 (ACT1) (Rutgeerts et al., 2005)	worldwide	121 (78/43)	42.4 ± 14.3	8.5 ± 1.7	Infliximab	0	0
ACT2 (Rutgeerts et al., 2005)	worldwide	121 (76/45)	40.5 ± 13.1	8.3 ± 1.5	Infliximab	0	0
Jiang 2015 (Jiang et al., 2015)	China	41 (26/15)	34.3 ± 14.3	6.0 ± 1	Infliximab	0	0
Reinisch 2011 (Reinisch et al., 2011)	North America /Europe	130 (83/47)	36.5 (18–75)	8.8 ± 1.61	Adalimumab	0	0
Sandborn 2012 (Sandborn et al., 2012)	North America/Europe/Australia/New Zealand/Israel	248 (142/106)	39.6 ± 12.47	8.9 ± 1.50	Adalimumab	40.3	NR
Suzuki 2014 (Suzuki et al., 2014)	Japan	90 (61/29)	42.5 ± 14.6	8.6 ± 1.4	Adalimumab	0	0
Sandborn 2014 (PURSUIT-SC) (Sandborn et al., 2014b)	Eastern Europe/North America/Asia Pacific/South Africa/Western Europe/Israel	331 (151/180)	40.0 ± 13.54	8.6 ± 1.53	Golimumab	0	0
PURSUIT-Maintenance (Sandborn et al., 2014a)		154 (65/89)	39.1 ± 13.11	8.5 ± 1.34	Golimumab	0	0
Hibi 2017 (Hibi et al., 2017)	Japan	32 (19/13)	39.30 ± 12.00	8.0 (6; 11)	Golimumab	0	0
Sandborn2020 (Sandborn et al., 2020)	worldwide	54 (31/23)	41.6 ± 14.1	9.0 (6–12)	Vedolizumab	38.9^b^	NR
Feagan 2013 (Feagan et al., 2013)	worldwide	225 (132/93)	40.1 ± 13.1	8.5 ± 1.8	Vedolizumab	48.2	41.0
Motoya 2019 (Motoya et al., 2019)	Japan	164 (99/65)	42.3 ± 14.4	8.3 ± 1.5	Vedolizumab	51.4	50.1
VARSITY 2019 (Sands et al., 2019a)	34 countries	386 (216/170)	40.5 ± 13.4	8.7 ± 1.5	Adalimumab	79.0	79.5
​	​	385 (234/151)	40.8 ± 13.7	8.7 ± 1.6	Vedolizumab	79.2	81.3
Sands 2019 (Sands et al., 2019b)	worldwide	322 (195/127)	41.7 ± 13.7	8.9 ± 1.5	Ustekinumab	53.9	50.5
D’Haens 2023 (D’Haens et al., 2023)	34 countries	868 (338/530)	42.9 ± 13.9	NA	Mirikizumab	NR	36.32
Rubin 2025 (Rubin et al., 2025)	32 countries	421 (183/238)	41.0 ± 13.9	9.1 ± 1.4	Guselkumab	49.1	42.9
​	​	188 (86/102)	40.3 ± 13.0	9.0 ± 1.4	Guselkumab	42.3	37.2
Louis E2024 (Louis et al., 2024)	41 countries	650 (265/385)	41.8 ± 13.5	7.1 ± 1.2	Risankizumab	NR	52
​	​	179 (74/105)	40.9 ± 14.7	7.2 ± 1.2	Risankizumab	NR	75
Sandborn2017 (Sandborn et al., 2017)	worldwide	476 (277/199)	41.3 ± 14.1	9.0 ± 1.4	Tofacitinib	46.7	48.7
​	​	429 (259/170)	41.1 ± 13.5	9.0 ± 1.5	Tofacitinib	44.7	47.9
​	​	198 (103/95)	41.9 ± 13.7	3.3 ± 1.8	Tofacitinib	52.3	55.3
Danese2022 (Danese et al., 2022a)	39 countries	319 (198/121)	43.0 ± 23.0	7.0 ± 1.2	Upadacitinib	54.12	52.0
​	40 countries	341 (214/127)	40.0 ± 24.0	7.0 ± 1.2	Upadacitinib	51.7	52.7
​	35 countries	148 (95/53)	40.0 ± 22.0	7.0 ± 1.2	Upadacitinib	51.9	49.9
Feigen 2021 (Feagan et al., 2021)	40 countries	245 (122/123)	42 ± 13.1	8.6 ± 1.3	Filgotinib	51.1	46.7
Sandborn2021 (Sandborn et al., 2021)	30 countries	429 (245/184)	41.4 ± 13.5	8.9 ± 1.5	Ozanimod	34.3	34.5
Severine2022 (Vermeire et al., 2022)	15 countries	108 (48/60)	36 (18–77)	8.48 ± 1.36	Etrolizumab	0	0
Danese 2022 (Danese et al., 2022b) (GARDENIA)	19 countries	199 (81/118)	37 (18–76)	8.6 ± 1.53	Etrolizumab	0	0
Rubin2022 (Rubin et al., 2022)	worldwide	144 (70/74)	36.5 (18–79)	8.9 ± 1.3	Etrolizumab	0	0
​	​	144 (70/74)	36.5 (18–79)	8.9 ± 1.3	Etrolizumab	0	0
Sandborn2023 (ELEVATE UC 52) (Sandborn et al., 2023)	40 countries	289 (137/152)	41.2 ± 14.0	9.0 ± 1.5	Etrasimod	37.6	NR
ELEVATE UC 12 (Sandborn et al., 2023)	​	238 (103/135)	40.3 ± 13.5	8.7 ± 1.5	Etrasimod	37.3	NR
Naganuma 2025 (Naganuma et al., 2025)	Japan	33 (9/24)	37.0 (26.5, 50.5)	9.0 (8.0, 9.0)	Infliximab	0	0
​	​	34 (14/20)	50.5 (33.8, 67.8)	9.0 (8.0, 9.0)	Vedolizumab	0	0
​	​	30 (13/17)	43.0 (30.0, 56.3)	8.5 (7.5, 9.0)	Ustekinumab	0	0

Abbreviations: F/M, female/male; TNF, tumor necrosis factor; NR, not reported.

^a^ Data were extracted from the original trial’s baseline characteristics table. A discrepancy exists in the original publication, where the subgroup analysis text reports 80 patients (37.0%) in the full analysis set.

^b^ For trials that only reported the proportion of patients with prior anti-TNF failure without specifying the total biologic-experienced population, the anti-TNF failure rate was utilized as a conservative proxy for prior biologic exposure.

### 3.3 Risk of bias assessment

The overall risk of bias across the included studies was low (95.8%). The summary of the risk of bias is presented in Figure 2, and the detailed assessment for each study is provided in Supplementary Table S3. Notably, the study by Naganuma et al. (2025) was assessed as having “some concerns” in the domain of “deviations from intended interventions” due to its open-label design. However, given that the endoscopic endpoints utilized in this study were objective and centrally reviewed, and that they provided the only key direct evidence comparing Infliximab, Vedolizumab, and Ustekinumab, the trial was retained in the primary network analysis. Furthermore, Assessments of global heterogeneity ( $\tau^{2}$ ) and consistency ( ΔDIC ) confirmed the mathematical robustness of the network models for all outcomes (Supplementary Table S2).

**FIGURE 2:**
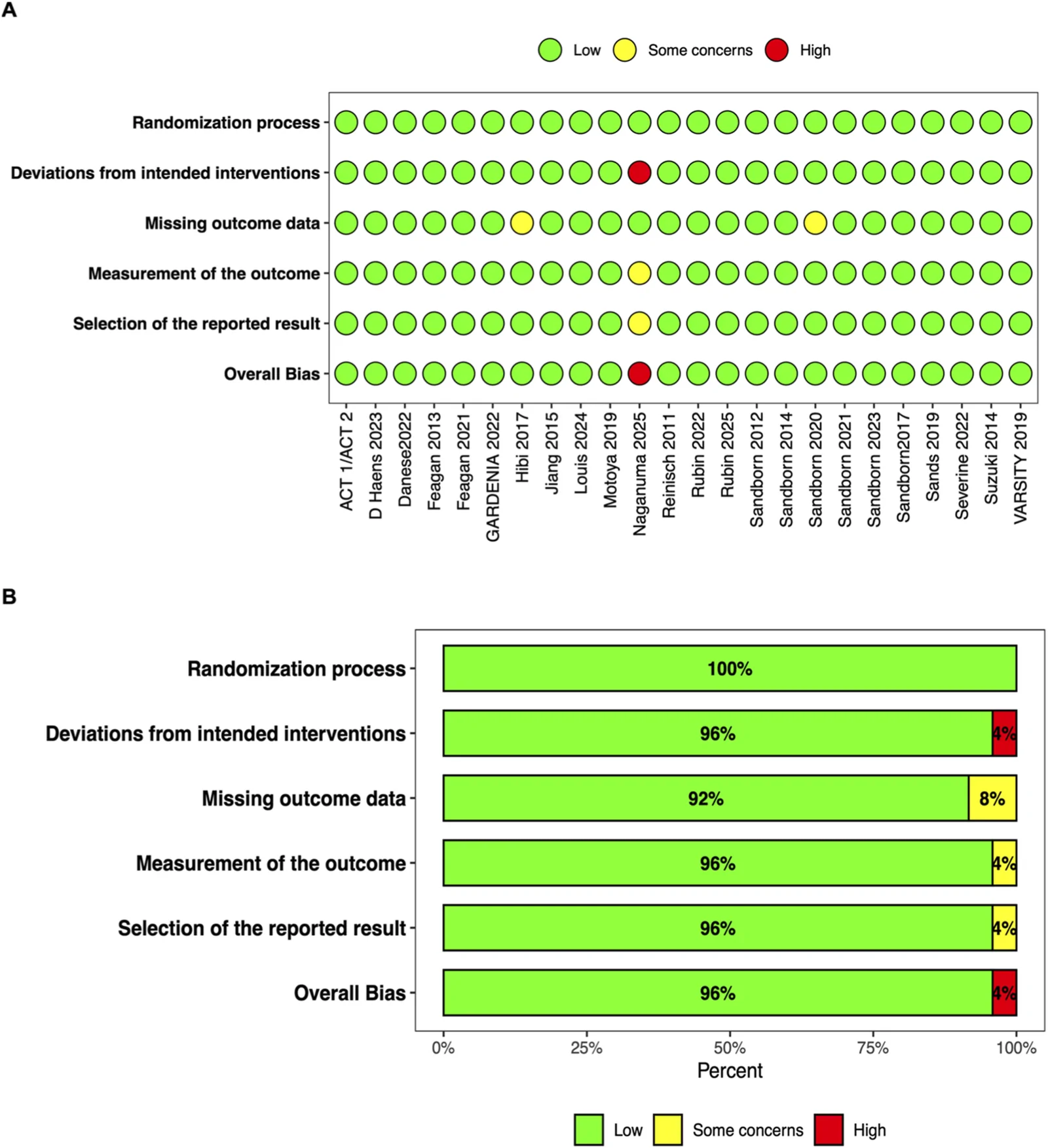
Risk of bias assessment of included randomized controlled trials. **(A)** The traffic light plot presents the domain-level judgments for each individual study. **(B)** Summary bar chart presents the proportion of studies at each level of risk for each bias domain. Risk of bias was evaluated at the study level (n = 24) rather than the publication level (n = 25), as multiple reports belonging to the same trial (e.g., the golimumab induction and maintenance studies) were assessed collectively.

### 3.4 Efficacy outcomes

Before the comparative analysis, the consistency of the network was evaluated. Both the global inconsistency test (*P* > 0.05) and the node-splitting method indicated no significant deviation between direct and indirect evidence, ensuring the reliability of the subsequent synthesis.

#### 3.4.1 Network structure and characteristics

In this study, 15 nodes were included in the induction stage and 16 nodes in the maintenance stage (the latter distinguishing between 15 and 30 mg doses of Upadacitinib), forming a placebo-centered star topology network (Figure 3). The direct head-to-head evidence was relatively limited and mainly concentrated among traditional biological agents. The key evidence loop was composed of comparative tests of Infliximab, Vedolizumab, and Ustekinumab, incorporating recent results from Naganuma et al. and the VARSITY trial. For novel small molecule drugs (Upadacitinib, Ozanimod) and IL-23 inhibitors (Risankizumab, Guselkumab), the comparisons depended entirely on indirect comparison through placebo nodes.

**FIGURE 3:**
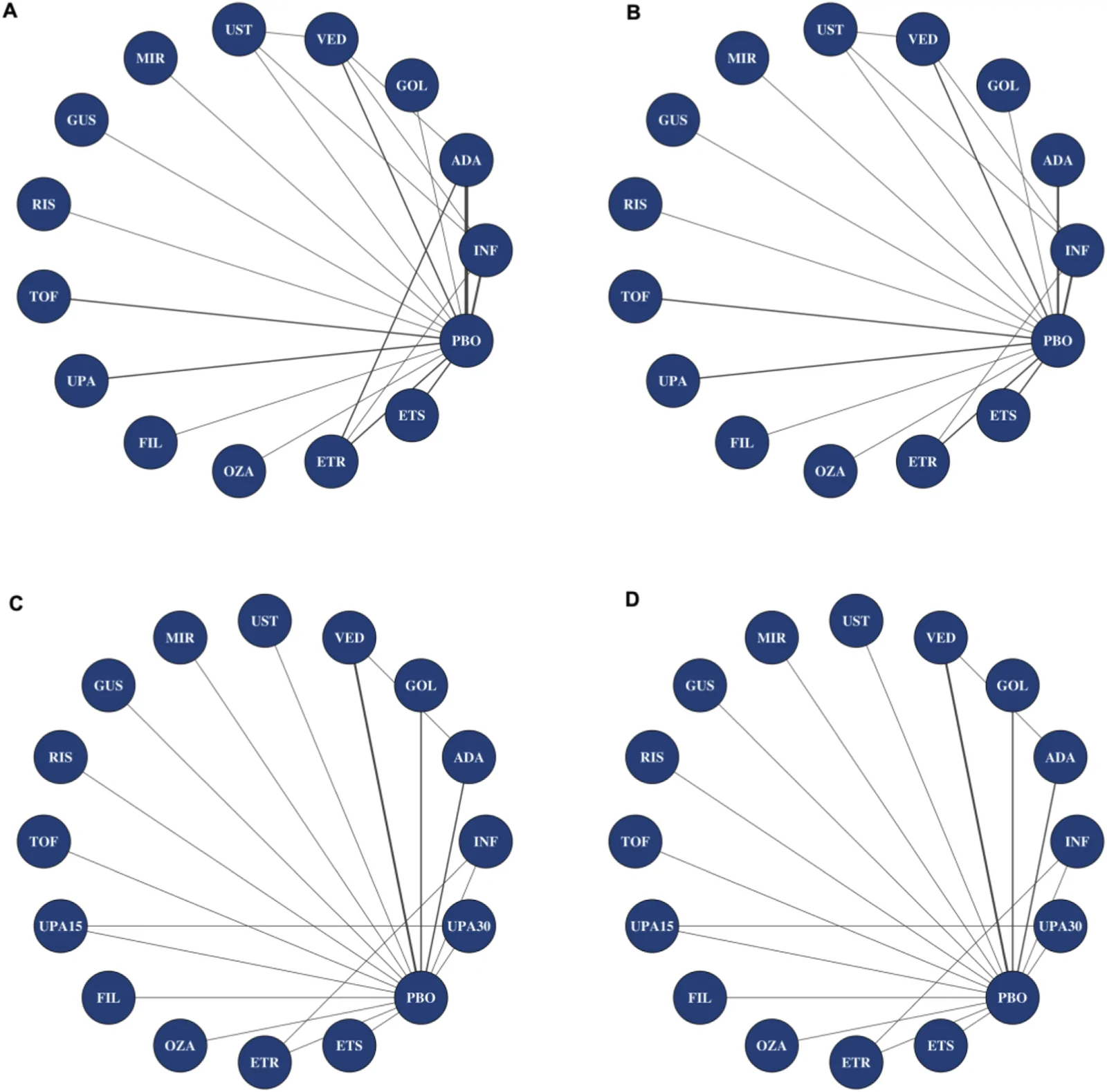
Network plots of treatment comparisons for primary efficacy outcomes. **(A)** Clinical remission in the induction phase. **(B)** Endoscopic improvement in the induction phase. **(C)** Clinical remission in the maintenance phase. **(D)** Endoscopic improvement in the maintenance phase. The networks demonstrate a predominantly placebo-centered star topology. The induction networks **(A,B)** comprise 15 treatment nodes. The maintenance networks **(C,D)** consist of 16 nodes, as Upadacitinib was evaluated at two distinct maintenance doses (15 mg and 30 mg). Abbreviations: ADA, Adalimumab; ETRA, Etrasimod; ETRO, Etrolizumab; FIL, Filgotinib; GOL, Golimumab; GUS, Guselkumab; IFX, Infliximab; MIR, Mirikizumab; OZA, Ozanimod; PBO, Placebo; RIS, Risankizumab; TOF, Tofacitinib; UPA, Upadacitinib; UPA15, Upadacitinib 15 mg; UPA30, Upadacitinib 30 mg; UST, Ustekinumab; VED, Vedolizumab

#### 3.4.2 Comparative efficacy: induction phase

Clinical remission: All active interventions demonstrated statistical superiority over placebo. Upadacitinib (OR: 8.53; 95% CrI: 4.64–16.44) and Risankizumab (OR: 3.65; 95% CrI: 1.96–6.95) exhibited the most pronounced effect sizes (Figure 4A). In indirect head-to-head comparisons, Upadacitinib was significantly superior to Adalimumab, Vedolizumab, and Tofacitinib. Notably, no statistically significant difference was observed between upadacitinib and risankizumab for this endpoint.

**FIGURE 4:**
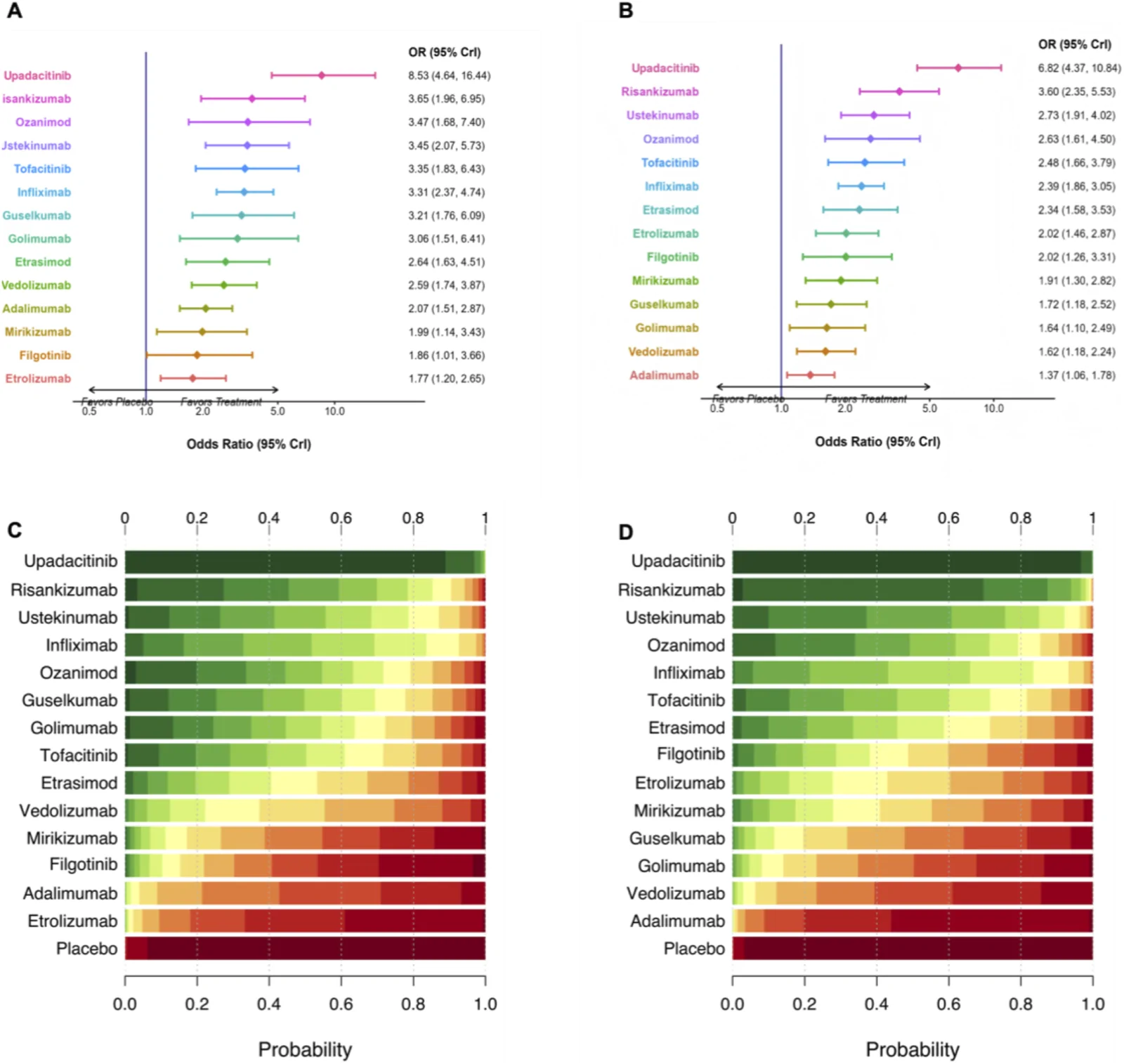
Comparative efficacy and probabilistic rankings of active interventions during the induction phase. Forest plots displaying ORs and 95% CrIs versus placebo for **(A)** clinical remission and **(B)** endoscopic improvement (OR > 1 indicates superior efficacy). Heatmaps and SUCRA rankings for **(C)** clinical remission and **(D)** endoscopic improvement. Heatmap colors denote rank probabilities (dark green = most efficacious [Rank 1]; dark red = least efficacious). Abbreviations: CrI, Credible Interval; OR, Odds Ratio; SUCRA, Surface Under the Cumulative Ranking curve. SUCRA rankings reflect probabilistic trends and must be interpreted alongside ORs and CrIs.

Endoscopic improvement: The efficacy hierarchy aligned with remission data yet displayed sharper stratification. Upadacitinib (OR vs. placebo: 6.73; 95% CrI: 4.42–10.79) ranked highest, demonstrating significant superiority over other active treatments including adalimumab, vedolizumab, and risankizumab (Figure 4B).

Probabilistic ranking (SUCRA): Upadacitinib dominated induction, placing first for both clinical remission (SUCRA: 99.9%) and endoscopic improvement (SUCRA: 99.9%), while Risankizumab held a clear second ahead of Ustekinumab (Figures 4C,D). Meanwhile, legacy biologics such as adalimumab slipped markedly and proved superior only to placebo for endoscopic improvement.

#### 3.4.3 Comparative efficacy: maintenance phase

Clinical remission: Etrasimod (OR: 5.88; 95% CrI: 2.26–16.49) ranked highest, followed by upadacitinib 30 mg (OR: 5.79; 95% CrI: 2.43–13.74) and filgotinib (OR: 5.69; 95% CrI: 1.45–27.35), though wide intervals for etrasimod and filgotinib suggest uncertainty due to limited trials. Other JAK inhibitors demonstrated robust efficacy, including upadacitinib 15 mg (OR: 4.38; 95% CrI: 1.78–10.49) and tofacitinib (OR: 4.06; 95% CrI: 1.67–10.07). Six other agents (including golimumab, vedolizumab, and guselkumab) maintained superiority over placebo, whereas ozanimod, ustekinumab, risankizumab, and etrolizumab did not reach statistical significance (Figure 5A).

**FIGURE 5:**
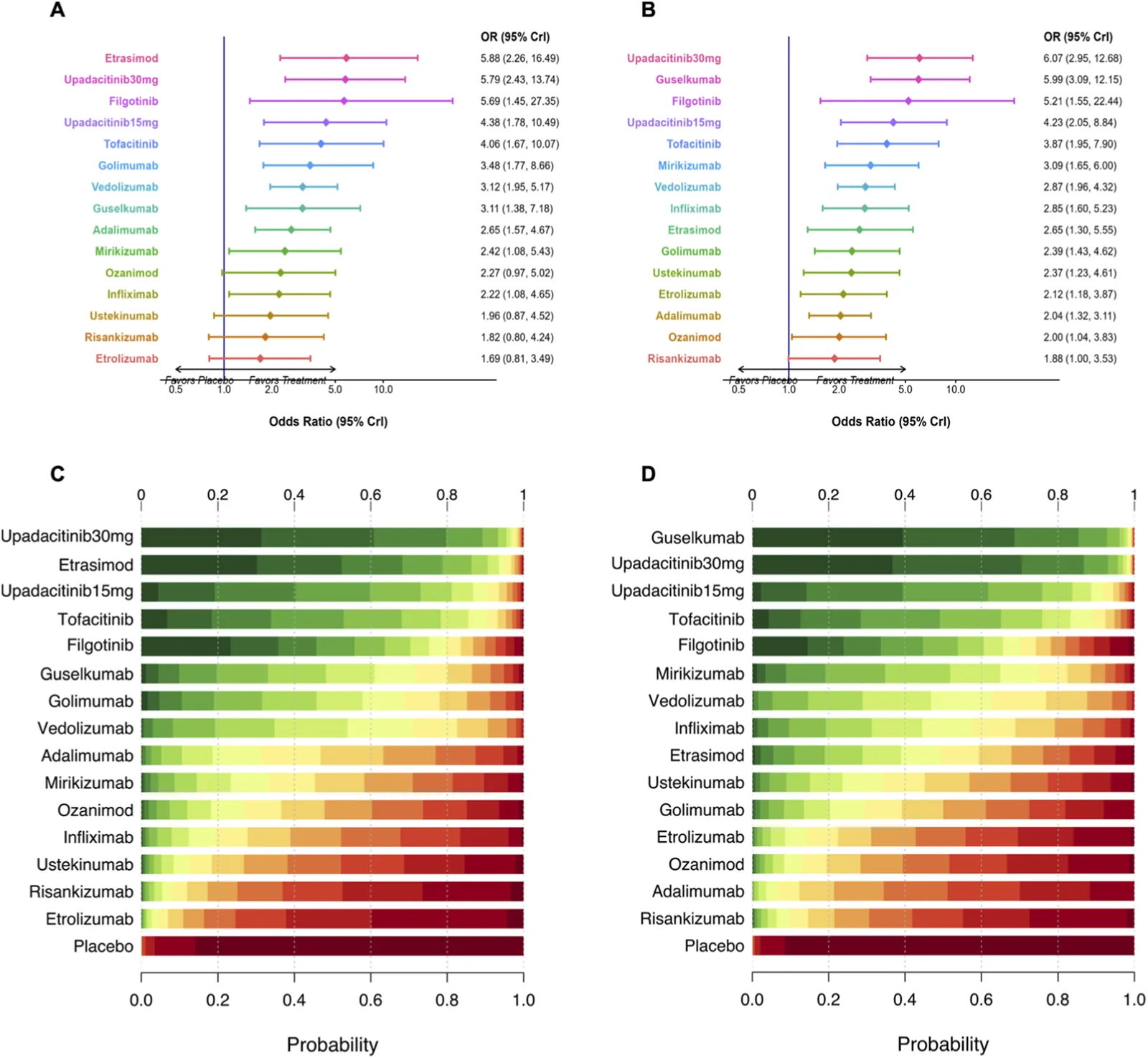
Comparative efficacy and probabilistic rankings of active interventions during the maintenance phase. Forest plots displaying ORs and 95% CrIs versus placebo for **(A)** clinical remission and **(B)** endoscopic improvement (OR > 1 indicates superior efficacy). Heatmaps and SUCRA rankings for **(C)** clinical remission and **(D)** endoscopic improvement. Heatmap colors denote rank probabilities (dark green = most efficacious [Rank 1]; dark red = least efficacious). Abbreviations: CrI, Credible Interval; OR, Odds Ratio; SUCRA, Surface Under the Cumulative Ranking curve. SUCRA rankings reflect probabilistic trends and must be interpreted alongside ORs and CrIs.

Endoscopic improvement: In the analysis of mucosal healing, the efficacy hierarchy was co-led by JAK inhibitors and IL-23 antagonists (Figure 5B). Upadacitinib 30 mg (OR: 6.07; 95% CrI: 2.95–12.68) and guselkumab (OR: 5.99; 95% CrI: 3.09–12.15) exhibited the highest efficacy estimates against placebo, both demonstrating highly stable results. Other therapies, including filgotinib (OR: 5.21; 95% CrI: 1.55–22.44) and the IL-23 inhibitor mirikizumab (OR: 3.09; 95% CrI: 1.65–6.00), also demonstrated significant efficacy, though filgotinib displayed a wider credible interval. Furthermore, upadacitinib 30 mg demonstrated statistical superiority over adalimumab, but not ustekinumab in indirect head-to-head comparisons.

Probabilistic ranking (SUCRA): For clinical remission, Upadacitinib 30 mg achieved the highest ranking (SUCRA: 92.6%), followed closely by the S1P modulator Etrasimod (SUCRA: 89.1%) and Upadacitinib 15 mg (Figure 5C). For endoscopic improvement, Guselkumab emerged as the top-ranked agent (SUCRA: 95%), marginally outperforming Upadacitinib 30 mg (SUCRA: 94.9%). This divergence, visualized in Figure 5D, highlights the exceptional capacity of IL-23 antagonists to induce deep mucosal healing. In contrast, established drugs such as Adalimumab and Risankizumab were ranked at a lower level in this maintenance treatment indicator.

### 3.5 Safety and tolerability outcomes

#### 3.5.1 Tolerability outcome (discontinuation due to adverse events)

Induction phase: Ustekinumab demonstrated the most favorable tolerability, ranking first (SUCRA: 99.0%), and was the only intervention significantly reducing the risk of discontinuation due to adverse events versus placebo (OR: 0.00; 95% CrI: 0.00–0.08). Comparisons for all other active interventions lacked statistical significance (e.g., Risankizumab: OR 0.15, 95% CrI: 0.01–2.65), likely due to the low overall adverse event incidence during this short phase. Consequently, while SUCRA generated a probabilistic hierarchy—positioning Risankizumab (74.4%), Mirikizumab (70.6%), and Upadacitinib (68.9%) higher, and Etrasimod (8.3%) lower—these rankings represent numerical trends rather than definitive stratifications, given the wide credible intervals and large statistical uncertainty (Figures 6A,C).

**FIGURE 6:**
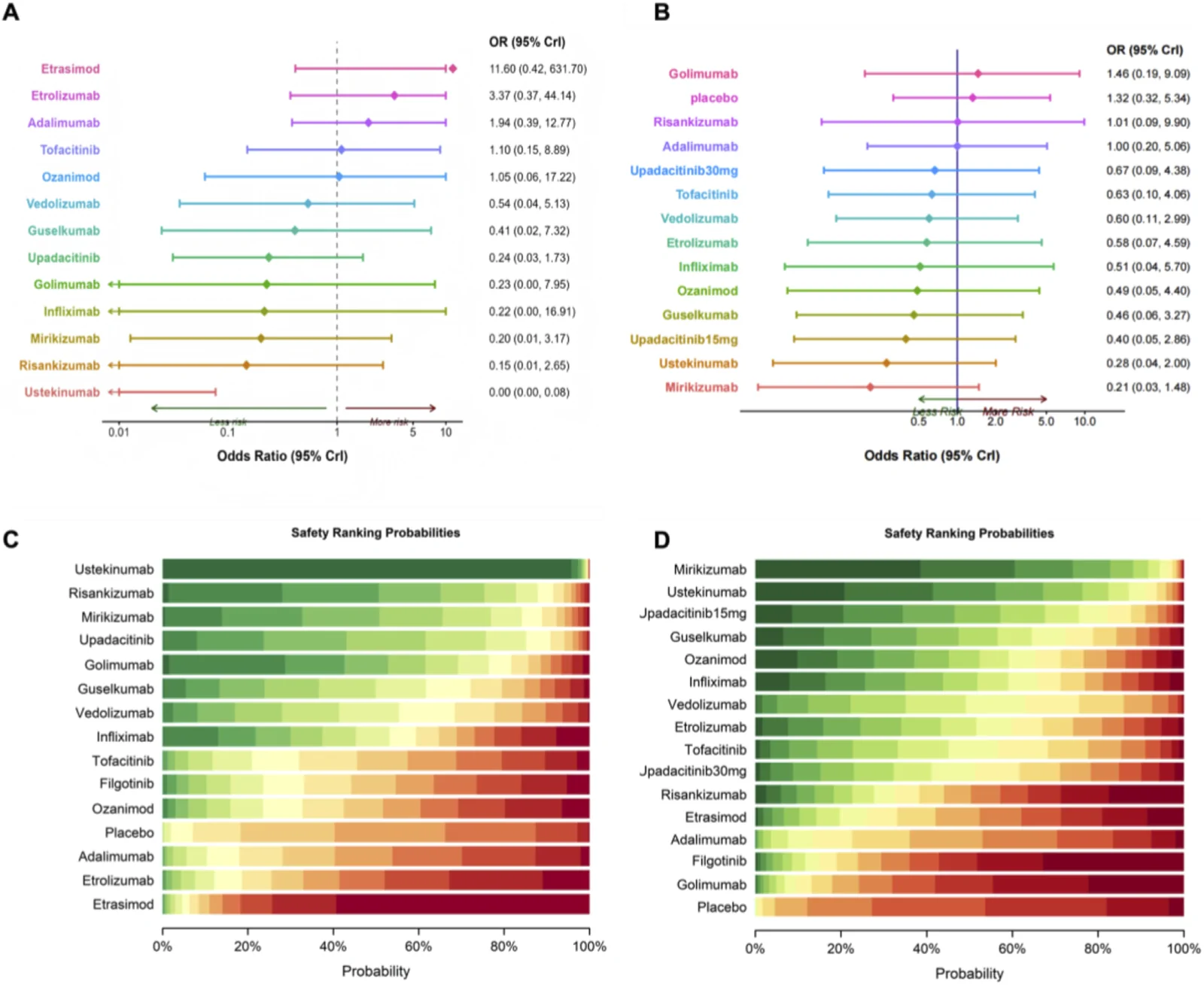
Comparative tolerability (discontinuation due to adverse events) of active interventions. **(A)** Forest plot illustrating the odds ratios (ORs) and 95% credible intervals (CrIs) for discontinuations due to adverse events compared with placebo during the induction phase. **(B)** Forest plot illustrating ORs and 95% CrIs for discontinuations due to adverse events during the maintenance phase. **(C)** Heatmap and SUCRA tolerability rankings for the induction phase. **(D)** Heatmap and SUCRA tolerability rankings for the maintenance phase. Tolerability is evaluated based on the rate of treatment discontinuations due to AEs; an OR < 1 indicates a lower risk of discontinuation compared to placebo. The SUCRA bar charts **(C,D)** are color-coded to denote tolerability rankings (green = most tolerable; red = least tolerable). Higher SUCRA scores denote a higher probability of being the most tolerable treatment. Abbreviations: AEs, adverse events; CrI, credible interval; OR, odds ratio; SUCRA, surface under the cumulative ranking curve. SUCRA rankings reflect probabilistic trends and must be interpreted alongside ORs and 95% CrIs.

Maintenance phase: IL-23 antagonists demonstrated superior tolerability (Figures 6B,D). Mirikizumab ranked highest (SUCRA: 87.9%), followed by Ustekinumab (80.4%) and Upadacitinib 15 mg (70.4%). Notably, Mirikizumab (OR: 0.16; 95% CrI: 0.04–0.63), Ustekinumab (OR: 0.22; 95% CrI: 0.05–0.85), and Vedolizumab (OR: 0.46; 95% CrI: 0.20–0.99) significantly reduced the risk of adverse event-related discontinuations versus placebo. Although Guselkumab (64.1%) and Upadacitinib 15 mg ranked favorably, their comparisons to placebo were not statistically significant. Conversely, Upadacitinib 30 mg (50.3%) occupied the intermediate tier, suggesting a dose-dependent tolerability trade-off compared to its 15 mg counterpart. Filgotinib ranked lower (21.3%), indicating a relatively weaker safety profile among small molecules. As in the induction phase, hierarchical differences among agents lacking statistical significance relative to placebo must be strictly interpreted as numerical trends.

#### 3.5.2 General safety overview (SAEs and any AEs)

The analysis of any adverse event (AEs) showed that there was no statistically significant difference between the treatment groups compared with placebo, which may reflect the higher background activity of the disease in the placebo group (Supplementary Tables S5, S6).

Regarding serious adverse events (SAEs), there was very little significant difference. During the induction phase, only Risankizumab (OR: 0.23; 95% CrI: 0.10–0.54) and Ustekinumab (OR: 0.47; 95% CrI: 0.19–0.99) significantly reduced the risk of SAE compared with placebo. During the maintenance phase, no active treatment is significantly different from placebo. Estimates for agents such as Filgotinib were unstable due to zero event counts in the control group. Therefore, in the subsequent efficacy-tolerability cluster analysis, discontinuation due to adverse events were prioritised as the main indicator (Supplementary Tables S6, S7).

### 3.6 Efficacy-tolerability clustered ranking analysis

In order to visually present the efficacy-tolerability trade-off of the treatment plan, we conducted a cluster ranking analysis and compared the efficacy indicators with tolerability.

Induction stage (Figures 7A,B): Treatments exhibited distinct stratification and intra-class heterogeneity. Ustekinumab, Upadacitinib, and Risankizumab co-occupied the most favorable quadrant with varying strengths: Upadacitinib demonstrated the highest efficacy, whereas Ustekinumab and Risankizumab maximized tolerability while maintaining robust efficacy. Infliximab also clustered in this favorable region, reflecting its rapid onset of action. Conversely, intra-class temporal differences emerged among IL-23 antagonists: Mirikizumab and Guselkumab clustered in moderate regions during the induction phase, which may suggest a relatively slower onset of action compared to Risankizumab, despite their known long-term efficacy. Etrasimod and Etrolizumab positioned in the least favorable quadrant due to suboptimal rankings across both dimensions.

**FIGURE 7:**
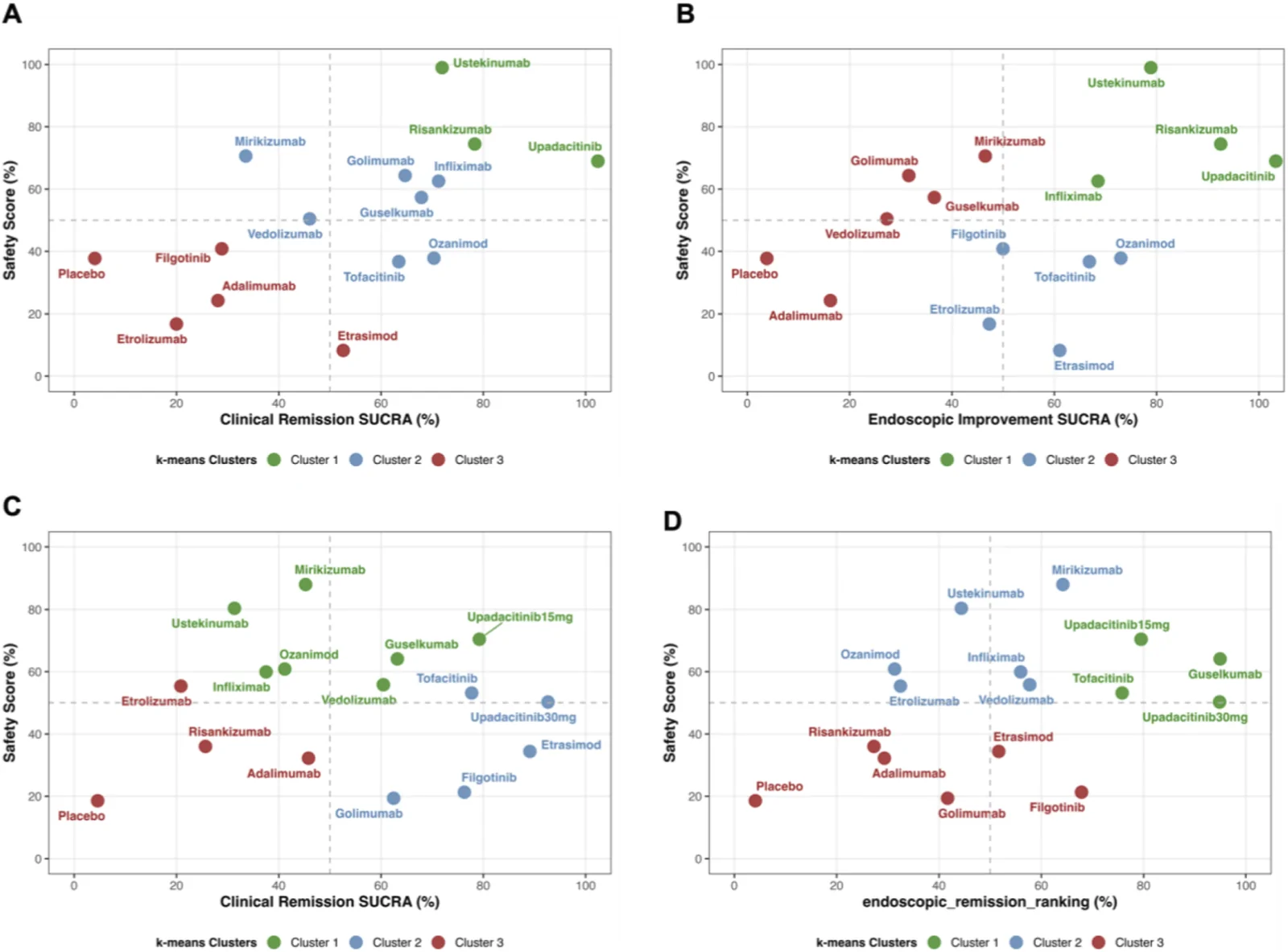
Clustered ranking analysis of efficacy versus tolerability for active interventions. Bivariate clustered ranking plots mapping SUCRA values for efficacy (x-axis) against tolerability (y-axis; discontinuation due to adverse events). Plots display **(A)** clinical remission and **(B)** endoscopic improvement during induction, alongside **(C)** clinical remission and **(D)** endoscopic improvement during maintenance. Treatments were objectively grouped into three clusters (Green, Blue, Red) via a k -means algorithm based on Euclidean distances. Crucially, these color-coded clusters represent strictly data-driven mathematical groupings rather than subjective hierarchical rankings. Dashed lines at 50% serve purely as visual references. The upper-right direction indicates the most favorable efficacy-tolerability profile (highest cumulative probabilities for both efficacy and tolerability).

Maintenance stage (Figures 3C,D): For clinical remission versus tolerability (Figure 3C), IL-23 antagonists (Guselkumab, Mirikizumab) and Upadacitinib 15 mg occupied the most favorable quadrant, balancing high efficacy with excellent tolerability. Notably, Mirikizumab and Guselkumab consistently peaked on the tolerability axis, underscoring the long-term tolerability advantages of IL-23 inhibitors.

Analyses of endoscopic improvement (Figure 3D) revealed a similar pattern. Upadacitinib 30 mg positioned furthest right, indicating maximal mucosal healing; however, its moderate tolerability ranking highlighted a dose-dependent trade-off compared to the 15 mg dose. Filgotinib demonstrated significant efficacy but fell into the lower-right quadrant, suggesting robust therapeutic effects coupled with lower tolerability (potentially reflecting safety signals or poor patient retention).

### 3.7 Subgroup analysis: prior biologic exposure

Efficacy outcomes were stratified according to prior biologic exposure to evaluate differences between biologic-naïve and biologic-experienced populations. Biologic-Naïve Cohort: In the biologic-naïve population, all active drugs demonstrated statistical superiority over placebo during the induction phase (Supplementary Figures S1A,B). Among them, Ustekinumab emerged as the most effective option, ranking first in both clinical remission (OR: 5.26) and endoscopic improvement (OR: 8.86). However, the maintenance phase was characterized by greater statistical uncertainty due to wide credible intervals; notably, only Golimumab maintained a significant statistical advantage for clinical remission (OR: 5.31). Although endoscopic improvement showed positive trends across agents, no drug achieved statistically significant results in this specific phase (Supplementary Figures S1C,D).

Biologic-Exposed Cohort: In the biologic-exposed cohort, Upadacitinib dominated the induction landscape, exhibiting the greatest effect sizes for clinical remission (OR: 9.43) and endoscopic improvement (OR: 8.25), as shown in Supplementary Figures S2A,B. While most biologics retained efficacy, Filgotinib failed to achieve statistical significance for clinical remission in this subgroup. During the maintenance phase, Upadacitinib 30 mg continued to lead in clinical remission (OR: 7.87). Regarding endoscopic improvement (Supplementary Figure S2D), Guselkumab (OR: 9.33) demonstrated therapeutic efficacy comparable to Upadacitinib 30 mg (OR: 9.30), positioning both as highly efficacious alternatives for refractory cases. Conversely, Etrolizumab did not reach statistical significance for clinical remission in this subgroup.

### 3.8 Sensitivity analysis and risk of bias assessment

To verify the robustness of the primary findings, two sensitivity analyses were conducted. First, considering the potential open-label bias mentioned above, we excluded the study by Naganuma et al. from the network (Naganuma et al., 2025). The resulting clustering pattern remained consistent with the main analysis, confirming the superior performance of IL-23 antagonists. Second, a targeted sensitivity analysis, in which the HIBISCUS I and II trials were integrated into a single pooled node utilizing their original, un-imputed combined adalimumab data, preserved the relative efficacy hierarchy and validated the top ranking of Upadacitinib (Supplementary Table S8) (Rubin et al., 2022).

Comparison-adjusted funnel plots and Egger’s tests revealed no significant publication bias for most outcomes (P > 0.05), except for a slight asymmetry detected in maintenance endoscopic improvement (P = 0.0435, Supplementary Figure S3). As detailed in the discussion, this asymmetry is likely driven by small-study effects and trial-level heterogeneity rather than true publication bias, indicating that the impact on our overall conclusions remains negligible.

To ensure full transparency and avoid selective reporting, the complete probabilistic hierarchy and SUCRA metrics for all active interventions and placebo are comprehensively documented in Supplementary Table S9.

## 4 Discussion

### 4.1 Principal findings

This updated network meta-analysis delineates the comparative profiles of advanced therapies for moderate-to-severe ulcerative colitis, revealing distinct variations between efficacy and tolerability leaders. Upadacitinib demonstrated the highest ranking probabilities for clinical remission and endoscopic improvement across both phases. Conversely, regarding tolerability, Ustekinumab exhibited the most favorable profile during induction, whereas IL-23 antagonists emerged as the highly tolerable options during maintenance.

Efficacy-tolerability clustered ranking positioned IL-23 antagonists and low-dose Upadacitinib in the most favorable quadrant, balancing sustained efficacy with superior tolerability. In contrast, high-dose Upadacitinib and Filgotinib demonstrated robust therapeutic effects but lower tolerability rankings.

Subgroup analyses further refined these findings. In biologic-naïve patients, Ustekinumab and Upadacitinib provided the highest induction efficacy. For biologic-experienced cohorts, Upadacitinib remained the most efficacious; however, Guselkumab emerged as a highly effective biologic alternative for mucosal healing, with estimates comparable to high-dose JAK inhibitors. These findings remained robust across sensitivity and data imputation analyses, offering a quantitative evidence base to guide personalized treatment selection.

### 4.2 Efficacy hierarchy and mechanistic insights

This study clarifies the current treatment hierarchy, highlighting the efficacy of small molecules and the distinct therapeutic timing of IL-23 antagonists.

Upadacitinib consistently ranked highest for efficacy across both phases. This advantage likely derives from its rapid absorption, lack of immunogenicity (Shivaji et al., 2020), and broad-spectrum JAK inhibition. Its efficacy is durable (up to 96 weeks in U-ACTIVATE) and yields rapid improvements in mucosal healing and clinical symptoms like bowel urgency (Shehab et al., 2025). However, as these findings rely on indirect comparisons, head-to-head trials remain necessary to confirm definitive clinical superiority.

We also identified intra-class differences among IL-23 antagonists (Saab et al., 2025). Risankizumab excels during induction—suggesting rapid onset—whereas Guselkumab and Mirikizumab demonstrate superior maintenance efficacy. Guselkumab’s unique binding to CD64^+^ myeloid cells enables direct IL-23 neutralization at the mucosal source (Rubin et al., 2025), ikely driving its outstanding performance in biologic-experienced patients.

For refractory disease, this study updates previous paradigms. While earlier guidelines preferred Ustekinumab and Tofacitinib for biologic-experienced patients (Singh et al., 2024), current data shift this landscape. Upadacitinib now serves as the efficacy benchmark for biologic-experienced patients, with Guselkumab emerging as the leading biologic alternative. This supports a potential stratified strategy: considering broad-spectrum small molecules for prompt induction, followed by p19 inhibitors for tolerable, sustained maintenance. Importantly, this proposed sequence represents a clinical extrapolation rather than a direct conclusion derived from our network.

### 4.3 Clinical implications

While we acknowledge that these clinical implications are derived from indirect comparisons, these findings offer a possible clinical interpretation that supports moving beyond rigid “step-up” algorithms toward personalized medicine. For refractory disease, Upadacitinib emerges as a highly efficacious induction option, offering a higher probability of deep remission in biologic-exposed patients than recycling anti-TNF agents, consistent with the 2024 AGA guidelines (Singh et al., 2024), However, given the lower tolerability of JAK inhibitors, clinicians must carefully screen patients for thromboembolic risks and consider herpes zoster vaccination prior to treatment (Fudman et al., 2025). Conversely, for elderly or safety-focused patients, IL-23 antagonists (Guselkumab, Mirikizumab) and Ustekinumab represent highly suitable alternatives. Positioned among the most tolerable options in our clustered-ranking analysis, these agents show favourable tolerability relative to anti-TNF therapies according to our network meta-analysis. Importantly, selecting safer agents like Guselkumab no longer requires compromising significant efficacy. However, it is important to emphasize that while our clustered approach groups certain treatments together due to statistically similar bidimensional profiles, this does not establish clinically meaningful equivalence. Treatments within the same cluster may still differ substantially in absolute efficacy, specific adverse event risks, and the overall certainty of evidence. Therefore, these statistical clusters should aid in broad categorization rather than suggest direct clinical substitutability. Ultimately, these sequencing strategies and categorical groupings should be viewed as potential clinical pathways to facilitate shared clinical decision-making, rather than definitive treatment guidelines directly established by the present analysis.

Additionally, oral Upadacitinib avoids injection-related immunogenicity and administration inconvenience (Shivaji et al., 2020). For active patients lacking cardiovascular risks, these practical benefits further favor JAK inhibitors. Based on our analysis and external clinical guidelines, current evidence supports a tailored strategy: considering Upadacitinib to maximize remission in refractory cases, and utilizing IL-23 antagonists to optimize tolerability in vulnerable populations.

### 4.4 Strengths and limitations

Our study offers several strengths. As the most current network meta-analysis, it integrates trial data through 2025, including the latest results for Guselkumab, Mirikizumab, and long-term Upadacitinib data (U-ACTIVATE). Methodologically, to preserve network transitivity against standard 40–60 weeks trials, these extended 96-week endpoints were utilized as an exploratory reference rather than being blindly pooled, preventing the inflation of timepoint heterogeneity. This provides a contemporary and comprehensive efficacy ranking for the current therapeutic landscape. We performed clustered-ranking analysis to assess the efficacy-tolerability profiles of these agents within our broader benefit-risk framework, using adverse-event-related discontinuation as a tolerability surrogate. Importantly, although this metric provides a useful measure of tolerability, it does not capture the full safety profile of a given therapy. Our clustering analysis therefore reflects only the efficacy-tolerability trade-off and does not account for individual adverse events or rare, long-term safety outcomes. Drawing on the “clinical utility” concept proposed by Thorlund et al. (2014), these findings illustrate the trade-off between JAK inhibitors (high efficacy with lower tolerability) and the more balanced profile of IL-23 antagonists. Furthermore, strict stratification by prior biologic exposure addresses a key clinical gap, quantifying Upadacitinib and Guselkumab’s efficacy in refractory patients and directly supporting the 2024 AGA guidelines’ “second-line therapy” algorithms.

Despite these strengths, several limitations exist, beginning with the foundational assumption of transitivity in network meta-analysis. Because the majority of comparative efficacy estimates rely on indirect evidence due to the scarcity of direct head-to-head trials, clinical heterogeneity driven by variations in patient populations is a potential concern. However, a comprehensive review of key baseline characteristics—such as disease severity and the use of concomitant medications (Table 1)—demonstrated no systematic imbalances among the treatment nodes. Furthermore, to address the substantial heterogeneity potentially introduced by prior biologic exposure, we conducted rigorous stratified analyses. As detailed in Section 3.7, evaluating comparative efficacies specifically within the strictly defined biologic-naïve and biologic-exposed cohorts (Supplementary Figures S1, S2) ensured that variations in prior treatment history did not bias our indirect comparisons. Over a two-decade span, trial designs—such as “treat-through” versus “re-randomization” protocols—and endoscopic endpoint definitions have evolved significantly. Specifically, responder re-randomization trials might overestimate maintenance efficacy for certain drugs compared to standard treat-through designs, which could potentially introduce bias into our relative maintenance rankings. While consistency tests yielded reassuring results, the scarcity of direct head-to-head trials means the majority of comparative efficacy estimates rely on indirect evidence, requiring a nuanced evaluation of these findings.

The probabilistic nature of SUCRA and bivariate clustered ranking metrics introduces specific methodological constraints. Because these indices summarize ranking probabilities without reflecting the absolute magnitude or clinical relevance of treatment effects, marginal variations in effect estimates can trigger disproportionately large shifts in hierarchical position. Furthermore, for a comprehensive benefit-risk assessment, relative effect estimates (Odds Ratios) and treatment rankings are not necessarily sufficient to guide personalized clinical decision-making. While our analysis establishes a comparative hierarchy, it does not directly capture absolute differences in clinically relevant outcomes. Translating these relative benefits into absolute risk reductions depends on an individual patient’s baseline risk. Therefore, whether the observed between-group differences exceed established thresholds for clinical importance should be carefully evaluated in real-world clinical practice and future guideline development. Moreover, ranking probabilities fail to capture statistical uncertainty, such as the width of 95% credible intervals (CrIs), or the overall certainty of the underlying evidence, rendering the results potentially volatile when data are sparse. The reliance of these metrics on specific network structures underscores why clustered rankings should serve strictly as supplementary decision-making aids rather than definitive clinical directives, necessitating a parallel appraisal of point estimates (ORs) and their corresponding CrIs.

Parallel constraints apply to using discontinuation due to adverse events as a proxy for tolerability. These discontinuation rates are highly sensitive to trial-specific parameters, including varying study durations, distinct rescue therapy protocols, and specific withdrawal criteria. Subjective investigator judgment can also confound these metrics, as adverse event-related withdrawals occasionally capture early efficacy failure, such as disease exacerbation, rather than pure drug toxicity. This complicates the direct translation of tolerability hierarchies. Evaluating safety outcomes presents specific constraints due to inconsistent reporting and varying follow-up durations across trials. Furthermore, standard induction and maintenance trials often lack the sample sizes and extended observation periods required to detect rare but clinically important adverse events. Consequently, the absence of statistically significant differences in uncommon adverse events within our network should not be interpreted as definitive evidence of comparable safety. This vulnerability is mitigated when contextualized with extended follow-up data, such as the 96-week analysis of the U-ACTIVATE trial (Panaccione et al., 2025), demonstrating that the safety profiles remain stable over the long term. Finally, the predominantly North American and European geographic distribution, coupled with limited representation from Asia, South America, Australia, and Africa, may restrict the global applicability of our findings due to regional healthcare and demographic differences.

## 5 Conclusion

This network meta-analysis delineates a distinct therapeutic dichotomy in moderate-to-severe ulcerative colitis: based on current estimates, Upadacitinib consistently ranks at the top of the efficacy hierarchy, particularly for biologic-exposed patients, whereas IL-23 antagonists demonstrate excellent tolerability and well-balanced risks and benefits. The present results suggest moving beyond inflexible step-up regimens toward individualized, risk-stratified therapy tailored to each patient’s clinical profile. For patients with substantial inflammatory activity or treatment-refractory disease, upadacitinib may serve as a potent agent to achieve remission. In contrast, IL-23 antagonists constitute an attractive, well-tolerated choice for vulnerable patients and individuals with prominent safety concerns. Notably, the statistical rankings observed only reflect general probabilistic tendencies and should not be treated as definitive clinical rules. As our analysis lacks data on ultra-early treatment responses and subgroup analyses for patients with multiple prior treatment failures, prospective head-to-head trials are necessary to validate these quantitative rankings and establish robust clinical recommendations.

## Data Availability

The original contributions presented in the study are included in the article/Supplementary Material, further inquiries can be directed to the corresponding author.
