# Supplementary material for: Benefit-risk profiling of advanced therapies in moderate-to-severe ulcerative colitis: a clustered network meta-analysis
Source: Front Pharmacol. 2026 Oct 14;17:1961820. doi: 10.3389/fphar.2026.1961820 (PMC13713258; doi:10.3389/fphar.2026.1961820)
Supplement: Supplementary file 1 [file Supplementaryfile1.docx]

**the Cochrane Library Search History**

Search Name:	randomized controlled trial

Last Saved:	15/11/2025 15:20:17

Comment:

ID	Search

#1	MeSH descriptor: [Colitis, Ulcerative] explode all trees 2399

#2	(Colitis, Ulcerative):ti,ab,kw OR (Idiopathic Proctocolitis):ti,ab,kw OR (Inflammatory Bowel Disease):ti,ab,kw OR (Ulcerative Colitis Type):ti,ab,kw OR (Ulcerative Colitis):ti,ab,kw 10821

#3	(Colitis Gravis):ti,ab,kw 17

#4	#1 OR #2 OR #3 10831

#5	Upadacitinib:ti,ab,kw OR Rinvoq:ti,ab,kw OR Tofacitinib:ti,ab,kw OR Xeljanz:ti,ab,kw OR Filgotinib:ti,ab,kw OR Jyseleca:ti,ab,kw OR Ozanimod:ti,ab,kw OR Zeposia:ti,ab,kw OR Etrasimod:ti,ab,kw OR Infliximab:ti,ab,kw OR Remicade:ti,ab,kw OR Adalimumab:ti,ab,kw OR Humira:ti,ab,kw OR Golimumab:ti,ab,kw OR Simponi:ti,ab,kw OR Vedolizumab:ti,ab,kw OR Entyvio:ti,ab,kw OR Ustekinumab:ti,ab,kw OR Stelara:ti,ab,kw OR Mirikizumab:ti,ab,kw OR Risankizumab:ti,ab,kw OR Guselkumab:ti,ab,kw OR Tremfya:ti,ab,kw OR Etrolizumab:ti,ab,kw 12403

#6 #4 AND #5 2967

**PubMed** **Search History**

| Search number | Query | Results | Time | Date |
| --- | --- | --- | --- | --- |
| 4 | (("Colitis, Ulcerative"[Mesh] OR "Ulcerative Colitis"[Title/Abstract] OR "Idiopathic Proctocolitis"[Title/Abstract] OR "Colitis Gravis"[Title/Abstract]) AND ("Upadacitinib"[Title/Abstract] OR "Rinvoq"[Title/Abstract] OR "Tofacitinib"[Title/Abstract] OR "Xeljanz"[Title/Abstract] OR "Filgotinib"[Title/Abstract] OR "Jyseleca"[Title/Abstract] OR "Ozanimod"[Title/Abstract] OR "Zeposia"[Title/Abstract] OR "Etrasimod"[Title/Abstract] OR "Infliximab"[Title/Abstract] OR "Remicade"[Title/Abstract] OR "Adalimumab"[Title/Abstract] OR "Humira"[Title/Abstract] OR "Golimumab"[Title/Abstract] OR "Simponi"[Title/Abstract] OR "Vedolizumab"[Title/Abstract] OR "Entyvio"[Title/Abstract] OR "Ustekinumab"[Title/Abstract] OR "Stelara"[Title/Abstract] OR "Mirikizumab"[Title/Abstract] OR "Risankizumab"[Title/Abstract] OR "Guselkumab"[Title/Abstract] OR "Tremfya"[Title/Abstract] OR "Etrolizumab"[Title/Abstract])) AND ("randomized controlled trial"[Publication Type] OR "controlled clinical trial"[Publication Type] OR "randomized"[Title/Abstract] OR "placebo"[Title/Abstract] OR "clinical trials as topic"[MeSH Terms] OR "randomly"[Title/Abstract] OR "trial"[Title]) | 979 | 1:44:11 | 2025/11/15 |
| 3 | "randomized controlled trial"[Publication Type] OR "controlled clinical trial"[Publication Type] OR "randomized"[Title/Abstract] OR "placebo"[Title/Abstract] OR "clinical trials as topic"[MeSH Terms] OR "randomly"[Title/Abstract] OR "trial"[Title] | 1,840,961 | 1:43:39 | 2025/11/15 |
| 2 | "Upadacitinib"[Title/Abstract] OR "Rinvoq"[Title/Abstract] OR "Tofacitinib"[Title/Abstract] OR "Xeljanz"[Title/Abstract] OR "Filgotinib"[Title/Abstract] OR "Jyseleca"[Title/Abstract] OR "Ozanimod"[Title/Abstract] OR "Zeposia"[Title/Abstract] OR "Etrasimod"[Title/Abstract] OR "Infliximab"[Title/Abstract] OR "Remicade"[Title/Abstract] OR "Adalimumab"[Title/Abstract] OR "Humira"[Title/Abstract] OR "Golimumab"[Title/Abstract] OR "Simponi"[Title/Abstract] OR "Vedolizumab"[Title/Abstract] OR "Entyvio"[Title/Abstract] OR "Ustekinumab"[Title/Abstract] OR "Stelara"[Title/Abstract] OR "Mirikizumab"[Title/Abstract] OR "Risankizumab"[Title/Abstract] OR "Guselkumab"[Title/Abstract] OR "Tremfya"[Title/Abstract] OR "Etrolizumab"[Title/Abstract] | 33,375 | 1:43:13 | 2025/11/15 |
| 1 | "Colitis, Ulcerative"[Mesh] OR "Ulcerative Colitis"[Title/Abstract] OR "Idiopathic Proctocolitis"[Title/Abstract] OR "Colitis Gravis"[Title/Abstract] | 68,565 | 1:42:47 | 2025/11/15 |

**Embase**

Session Results

.......................................................

No. Query Results	Results Date

#9. #3 AND #4 AND #8	6,874 14 Nov 2025

#8. #5 OR #6 OR #7	2,668,419 14 Nov 2025

#7. ('clinical trials':ab,ti OR 'clinical trials as	4,193 14 Nov 2025

topic':ab,ti OR 'clinical trial':ab,ti) AND

topic:ab,ti AND [embase]/lim

#6. 'ulcerative colitis'/exp OR 'clinical trial	612,050 14 Nov 2025

(topic)'/exp

#5. 'randomized controlled trial':it OR 'controlled	1,933,123 14 Nov 2025

clinical trial':it OR randomized:ab,ti OR

placebo:ab,ti OR randomly:ab,ti OR trial:ti

#4. ('upadacitinib':ab,ti OR 'rinvoq':ab,ti OR	59,608 14 Nov 2025

'tofacitinib':ab,ti OR 'xeljanz':ab,ti OR

'filgotinib':ab,ti OR 'jyseleca':ab,ti OR

'ozanimod':ab,ti OR 'zeposia':ab,ti OR

'etrasimod':ab,ti OR 'infliximab':ab,ti OR

'remicade':ab,ti OR 'adalimumab':ab,ti OR

'humira':ab,ti OR 'golimumab':ab,ti OR

'simponi':ab,ti OR 'vedolizumab':ab,ti OR

'entyvio':ab,ti OR 'ustekinumab':ab,ti OR

'stelara':ab,ti OR 'mirikizumab':ab,ti OR

'risankizumab':ab,ti OR 'guselkumab':ab,ti OR

'tremfya':ab,ti OR 'etrolizumab':ab,ti) AND

[embase]/lim

#3. #1 OR #2	114,054 14 Nov 2025

#2. ('chronic ulcerative colitis':ab,ti OR 'colitis	79,858 14 Nov 2025

ulcerativa':ab,ti OR 'colitis ulcerosa':ab,ti OR

'colitis ulcerosa chronica':ab,ti OR 'colitis,

mucosal':ab,ti OR 'colitis, ulcerative':ab,ti OR

'colitis, ulcerous':ab,ti OR 'colon, chronic

ulceration':ab,ti OR 'histiocytic ulcerative

colitis':ab,ti OR 'mucosal colitis':ab,ti OR

'ulcerative colorectitis':ab,ti OR 'ulcerative

procto colitis':ab,ti OR 'ulcerative

proctocolitis':ab,ti OR 'ulcerous colitis':ab,ti

OR 'ulcerative colitis':ab,ti) AND [embase]/lim

#1. ('ulcerative colitis'/exp OR 'ulcerative	101,915 14 Nov 2025

colitis') AND [embase]/lim

.......................................................

**Supplementary Table S1** Literature search strategy and search strings

| Phase | Outcome | Heterogeneity: τ2 (95% CrI) | DIC (Consistency Model) | DIC (UME Inconsistency Model) | ΔDIC | Conclusion |
| --- | --- | --- | --- | --- | --- | --- |
| Induction | Clinical Remission | 0.0169 (0–0.1859) | 99.46 | 104.7 | 5.24* | Consistent |
|  | Endoscopic Improvement | 0.0064 (0–0.0792) | 88.34 | 91.62 | 3.28 | Consistent |
|  | Discontinuation due to AE | 0.5466 (0.0025–4.6779） | 73.75 | 73.99 | 0.24 | Consistent |
|  | Adverse Events (AEs) | 0.0178 (1e-04–0.1308) | 88.98 | 86.47 | 2.51 | Consistent |
|  | Serious AEs (SAEs) | 0.0643 (2e-04–0.5632) | 81.26 | 82.21 | 0.95 | Consistent |
| Maintenance | Clinical Remission | 0.022 (1e-04–0.4847) | 78.12 | 80.05 | 1.93 | Consistent |
|  | Endoscopic Improvement | 0.0272 (1e-04–0.4826) | 78.08 | 79.87 | 1.79 | Consistent |
|  | Discontinuation due to AE | 0.0765 (2e-04–1.5252) | 71.19 | 72.62 | 1.43 | Consistent |
|  | Adverse Events (AEs) | 0.2541 (0.0068–0.8152) | 65.24 | 65.3 | 0.06 | Consistent |
|  | Serious AEs (SAEs) | 0.4647 (0.0014–4.2862) | 68.22 | 68.11 | 0.11 | Consistent |

**Note:** * The ΔDIC value of 5.24 indicates a substantial difference in Deviance Information Criterion (ΔDIC > 5) favoring the consistency model over the unrelated mean effects (UME) inconsistency model, demonstrating an optimal model fit and supporting the global consistency assumption.

**Supplementary Table S2** Assessment of global heterogeneity and inconsistency using the Deviance Information Criterion (DIC) between consistency and UME models. Abbreviations: DIC, Deviance Information Criterion; UME, Unrelated Mean Effects; CrI, Credible Interval; AE, Adverse Event; SAE, Serious Adverse Event. A difference in DIC (ΔDIC) of less than 5 generally indicates no significant global inconsistency between the models

| Unique ID | Reviewer | Study ID | Reference | Aim | Effect of adhering to intervention? | Weight | Randomization process | Deviations from intended interventions | Missing outcome data | Measurement of the outcome | Selection of the reported result | Overall Bias |
| --- | --- | --- | --- | --- | --- | --- | --- | --- | --- | --- | --- | --- |
| 1 |  | ACT 1/ACT 2 |  | assignment to intervention (the 'intention-to-treat' effect) | NA | 2 | Low | Low | Low | Low | Low | Low |
| 2 |  | Jiang 2015 |  | assignment to intervention (the 'intention-to-treat' effect) | NA | 1 | Low | Low | Low | Low | Low | Low |
| 3 |  | Reinisch 2011 |  | assignment to intervention (the 'intention-to-treat' effect) | NA | 1 | Low | Low | Low | Low | Low | Low |
| 4 |  | Sandborn 2012 |  | assignment to intervention (the 'intention-to-treat' effect) | NA | 1 | Low | Low | Low | Low | Low | Low |
| 5 |  | Suzuki 2014 |  | assignment to intervention (the 'intention-to-treat' effect) | NA | 1 | Low | Low | Low | Low | Low | Low |
| 6 |  | Sandborn 2014 |  | assignment to intervention (the 'intention-to-treat' effect) | NA | 2 | Low | Low | Low | Low | Low | Low |
| 7 |  | Hibi 2017 |  | assignment to intervention (the 'intention-to-treat' effect) | NA | 1 | Low | Low | Some concerns | Low | Low | Low |
| 8 |  | Sandborn 2020 |  | assignment to intervention (the 'intention-to-treat' effect) | NA | 1 | Low | Low | Some concerns | Low | Low | Low |
| 9 |  | Feagan 2013 |  | assignment to intervention (the 'intention-to-treat' effect) | NA | 1 | Low | Low | Low | Low | Low | Low |
| 10 |  | Motoya 2019 |  | assignment to intervention (the 'intention-to-treat' effect) | NA | 1 | Low | Low | Low | Low | Low | Low |
| 11 |  | VARSITY 2019 |  | assignment to intervention (the 'intention-to-treat' effect) | NA | 1 | Low | Low | Low | Low | Low | Low |
| 12 |  | Sands 2019 |  | assignment to intervention (the 'intention-to-treat' effect) | NA | 1 | Low | Low | Low | Low | Low | Low |
| 13 |  | D’Haens 2023 |  | assignment to intervention (the 'intention-to-treat' effect) | NA | 1 | Low | Low | Low | Low | Low | Low |
| 14 |  | Rubin 2025 |  | assignment to intervention (the 'intention-to-treat' effect) | NA | 1 | Low | Low | Low | Low | Low | Low |
| 15 |  | Louis 2024 |  | assignment to intervention (the 'intention-to-treat' effect) | NA | 1 | Low | Low | Low | Low | Low | Low |
| 16 |  | Sandborn2017 |  | assignment to intervention (the 'intention-to-treat' effect) | NA | 3 | Low | Low | Low | Low | Low | Low |
| 17 |  | Danese2022 |  | assignment to intervention (the 'intention-to-treat' effect) | NA | 3 | Low | Low | Low | Low | Low | Low |
| 18 |  | Feagan 2021 |  | assignment to intervention (the 'intention-to-treat' effect) | NA | 1 | Low | Low | Low | Low | Low | Low |
| 19 |  | Sandborn 2021 |  | assignment to intervention (the 'intention-to-treat' effect) | NA | 1 | Low | Low | Low | Low | Low | Low |
| 20 |  | Severine 2022 |  | assignment to intervention (the 'intention-to-treat' effect) | NA | 1 | Low | Low | Low | Low | Low | Low |
| 21 |  | GARDENIA 2022 |  | assignment to intervention (the 'intention-to-treat' effect) | NA | 1 | Low | Low | Low | Low | Low | Low |
| 22 |  | Rubin 2022 |  | assignment to intervention (the 'intention-to-treat' effect) | NA | 1 | Low | Low | Low | Low | Low | Low |
| 23 |  | Sandborn 2023 |  | assignment to intervention (the 'intention-to-treat' effect) | NA | 2 | Low | Low | Low | Low | Low | Low |
| 24 |  | Naganuma 2025 |  | assignment to intervention (the 'intention-to-treat' effect) | NA | 1 | Low | High | Low | Some concerns | Some concerns | High |

**Supplementary Table S3** Detailed risk of bias assessment for each included study using the Cochrane RoB 2 tool

| IFX | ADA | GOL | VDZ | UST | MIRI | GUSE | RISA | TOFA | UPA | FIL | OZAN | ETRO | ETRA | PBO |
| --- | --- | --- | --- | --- | --- | --- | --- | --- | --- | --- | --- | --- | --- | --- |
| IFX | 1.20 (0.84, 1.71) | 1.24 (0.74, 2.05) | 1.09 (0.65, 1.76) | 1.48 (1.01, 2.56) | 1.27 (0.78, 2.07) | 1.21 (0.74, 2.02) | 1.53 (0.94, 2.53) | 1.24 (0.82, 1.91) | 1.08 (0.71, 1.66) | 1.31 (0.77, 2.23) | 1.13 (0.68, 1.92) | 1.34 (0.87, 2.09) | 1.20 (0.69, 2.10) | 1.22 (0.94, 1.61) |
| 0.83 (0.59, 1.19) | ADA | 1.04 (0.63, 1.68) | 0.91 (0.54, 1.48) | 1.23 (0.85, 2.12) | 1.06 (0.67, 1.70) | 1.01 (0.63, 1.62) | 1.28 (0.80, 2.03) | 1.04 (0.71, 1.53) | 0.90 (0.61, 1.31) | 1.10 (0.66, 1.84) | 0.95 (0.59, 1.54) | 1.12 (0.80, 1.56) | 1.00 (0.58, 1.69) | 1.02 (0.82, 1.27) |
| 0.81 (0.49, 1.34) | 0.97 (0.60, 1.59) | GOL | 0.88 (0.45, 1.59) | 1.18 (0.72, 2.36) | 1.02 (0.57, 1.84) | 0.98 (0.53, 1.80) | 1.23 (0.68, 2.23) | 1.01 (0.59, 1.70) | 0.87 (0.52, 1.48) | 1.05 (0.57, 2.01) | 0.91 (0.50, 1.69) | 1.08 (0.64, 1.90) | 0.97 (0.50, 1.85) | 0.98 (0.65, 1.51) |
| 0.91 (0.57, 1.54) | 1.10 (0.67, 1.86) | 1.13 (0.63, 2.21) | VDZ | 1.36 (0.81, 2.68) | 1.16 (0.64, 2.21) | 1.10 (0.62, 2.18) | 1.40 (0.79, 2.72) | 1.14 (0.66, 2.07) | 0.99 (0.59, 1.77) | 1.20 (0.67, 2.39) | 1.04 (0.57, 2.06) | 1.23 (0.71, 2.25) | 1.10 (0.58, 2.20) | 1.12 (0.72, 1.81) |
| 0.68 (0.39, 0.99) | 0.81 (0.47, 1.17) | 0.85 (0.42, 1.38) | 0.74 (0.37, 1.23) | UST | 0.87 (0.44, 1.37) | 0.83 (0.42, 1.34) | 1.05 (0.53, 1.66) | 0.85 (0.46, 1.29) | 0.73 (0.40, 1.11) | 0.90 (0.45, 1.50) | 0.77 (0.38, 1.27) | 0.91 (0.49, 1.43) | 0.81 (0.39, 1.40) | 0.83 (0.51, 1.12) |
| 0.79 (0.48, 1.28) | 0.94 (0.59, 1.49) | 0.98 (0.54, 1.75) | 0.86 (0.45, 1.56) | 1.15 (0.73, 2.27) | MIRI | 0.95 (0.54, 1.70) | 1.20 (0.69, 2.15) | 0.98 (0.58, 1.64) | 0.85 (0.51, 1.43) | 1.04 (0.56, 1.90) | 0.89 (0.50, 1.65) | 1.06 (0.62, 1.81) | 0.94 (0.50, 1.77) | 0.96 (0.64, 1.44) |
| 0.83 (0.50, 1.36) | 0.99 (0.62, 1.59) | 1.02 (0.56, 1.87) | 0.91 (0.46, 1.62) | 1.21 (0.74, 2.36) | 1.05 (0.59, 1.86) | GUSE | 1.26 (0.70, 2.26) | 1.03 (0.60, 1.74) | 0.89 (0.52, 1.50) | 1.09 (0.58, 1.99) | 0.94 (0.51, 1.68) | 1.11 (0.64, 1.90) | 1.00 (0.53, 1.84) | 1.01 (0.67, 1.54) |
| 0.66 (0.40, 1.07) | 0.78 (0.49, 1.25) | 0.81 (0.45, 1.47) | 0.72 (0.37, 1.27) | 0.96 (0.60, 1.89) | 0.83 (0.46, 1.46) | 0.79 (0.44, 1.44) | RISA | 0.81 (0.48, 1.35) | 0.70 (0.42, 1.18) | 0.86 (0.47, 1.59) | 0.74 (0.41, 1.33) | 0.88 (0.51, 1.50) | 0.78 (0.41, 1.47) | 0.80 (0.52, 1.21) |
| 0.81 (0.52, 1.22) | 0.96 (0.65, 1.40) | 0.99 (0.59, 1.69) | 0.88 (0.48, 1.51) | 1.18 (0.78, 2.16) | 1.02 (0.61, 1.71) | 0.97 (0.58, 1.66) | 1.23 (0.74, 2.07) | TOFA | 0.87 (0.56, 1.36) | 1.06 (0.60, 1.83) | 0.91 (0.54, 1.56) | 1.08 (0.66, 1.72) | 0.96 (0.54, 1.71) | 0.98 (0.71, 1.34) |
| 0.93 (0.60, 1.40) | 1.11 (0.76, 1.64) | 1.15 (0.67, 1.92) | 1.01 (0.57, 1.71) | 1.36 (0.90, 2.49) | 1.18 (0.70, 1.95) | 1.12 (0.67, 1.93) | 1.42 (0.85, 2.40) | 1.15 (0.74, 1.79) | UPA | 1.22 (0.70, 2.13) | 1.05 (0.62, 1.78) | 1.24 (0.78, 1.99) | 1.11 (0.62, 1.96) | 1.13 (0.83, 1.56) |
| 0.76 (0.45, 1.30) | 0.91 (0.54, 1.51) | 0.95 (0.50, 1.76) | 0.83 (0.42, 1.50) | 1.12 (0.67, 2.24) | 0.97 (0.53, 1.79) | 0.92 (0.50, 1.72) | 1.17 (0.63, 2.12) | 0.94 (0.55, 1.66) | 0.82 (0.47, 1.42) | FIL | 0.86 (0.46, 1.62) | 1.02 (0.57, 1.82) | 0.91 (0.47, 1.77) | 0.93 (0.59, 1.46) |
| 0.88 (0.52, 1.46) | 1.06 (0.65, 1.71) | 1.10 (0.59, 2.02) | 0.96 (0.49, 1.75) | 1.29 (0.79, 2.62) | 1.12 (0.61, 2.00) | 1.07 (0.59, 1.95) | 1.34 (0.75, 2.42) | 1.10 (0.64, 1.85) | 0.95 (0.56, 1.60) | 1.16 (0.62, 2.18) | OZAN | 1.19 (0.68, 2.08) | 1.06 (0.55, 2.04) | 1.08 (0.71, 1.66) |
| 0.74 (0.48, 1.16) | 0.89 (0.64, 1.25) | 0.92 (0.53, 1.57) | 0.81 (0.44, 1.41) | 1.10 (0.70, 2.02) | 0.95 (0.55, 1.62) | 0.90 (0.53, 1.56) | 1.14 (0.67, 1.95) | 0.93 (0.58, 1.51) | 0.81 (0.50, 1.29) | 0.98 (0.55, 1.75) | 0.84 (0.48, 1.48) | ETRO | 0.89 (0.49, 1.62) | 0.91 (0.64, 1.30) |
| 0.83 (0.48, 1.45) | 1.00 (0.59, 1.73) | 1.04 (0.54, 1.99) | 0.91 (0.45, 1.73) | 1.23 (0.71, 2.56) | 1.06 (0.56, 2.00) | 1.00 (0.54, 1.90) | 1.27 (0.68, 2.41) | 1.04 (0.59, 1.86) | 0.90 (0.51, 1.60) | 1.10 (0.57, 2.12) | 0.95 (0.49, 1.81) | 1.12 (0.62, 2.04) | ETRA | 1.02 (0.63, 1.67) |
| 0.82 (0.62, 1.07) | 0.98 (0.78, 1.22) | 1.02 (0.66, 1.55) | 0.90 (0.55, 1.38) | 1.21 (0.89, 1.97) | 1.04 (0.69, 1.56) | 0.99 (0.65, 1.50) | 1.26 (0.82, 1.91) | 1.02 (0.75, 1.40) | 0.89 (0.64, 1.21) | 1.08 (0.68, 1.68) | 0.93 (0.60, 1.42) | 1.10 (0.77, 1.56) | 0.98 (0.60, 1.58) | PBO |

**Supplementary Table S4** League table of any adverse events during the induction phase. Data are presented as odds ratios (ORs) with 95% confidence intervals (CIs). **Abbreviations:** ADA, adalimumab; ETRA, etrasimod; ETRO, etrolizumab; FIL, filgotinib; GOL, golimumab; GUSE, guselkumab; IFX, infliximab; MIRI, mirikizumab; OZAN, ozanimod; PBO, placebo; RISA, risankizumab; TOFA, tofacitinib; UPA, upadacitinib; UST, ustekinumab; VDZ, vedolizumab

| IFX | ADA | GOL | VDZ | UST | MIRI | GUSE | RISA | TOFA | UPA15 | FIL | OZAN | ETRO | ETRA | PBO | UPA30 |
| --- | --- | --- | --- | --- | --- | --- | --- | --- | --- | --- | --- | --- | --- | --- | --- |
| IFX | 0.53 (0.06, 4.21) | 0.40 (0.07, 2.16) | 0.63 (0.10, 3.82) | 0.70 (0.12, 4.78) | 0.70 (0.10, 4.65) | 0.64 (0.09, 4.29) | 0.69 (0.11, 4.72) | 0.60 (0.09, 4.31) | 0.58 (0.10, 4.02) | 0.51 (0.07, 3.41) | 0.42 (0.07, 2.81) | 0.96 (0.32, 2.91) | 0.41 (0.06, 2.80) | 0.62 (0.13, 2.93) | 0.57 (0.09, 4.00) |
| 1.89 (0.24, 15.67) | ADA | 0.77 (0.14, 3.66) | 1.19 (0.41, 3.54) | 1.35 (0.25, 8.15) | 1.32 (0.24, 7.91) | 1.21 (0.20, 7.02) | 1.32 (0.24, 7.56) | 1.14 (0.21, 6.79) | 1.10 (0.21, 6.83) | 0.96 (0.16, 5.82) | 0.79 (0.14, 4.44) | 1.83 (0.31, 10.97) | 0.78 (0.14, 4.78) | 1.18 (0.29, 4.73) | 1.09 (0.19, 6.12) |
| 2.52 (0.46, 14.89) | 1.30 (0.27, 7.14) | GOL | 1.55 (0.51, 5.37) | 1.74 (0.48, 7.34) | 1.71 (0.49, 7.52) | 1.56 (0.44, 6.96) | 1.72 (0.49, 7.29) | 1.50 (0.42, 6.54) | 1.45 (0.43, 6.30) | 1.25 (0.33, 5.23) | 1.03 (0.29, 4.44) | 2.39 (0.61, 9.90) | 1.01 (0.27, 4.31) | 1.54 (0.73, 3.88) | 1.43 (0.38, 6.21) |
| 1.60 (0.26, 9.91) | 0.84 (0.28, 2.47) | 0.64 (0.19, 1.96) | VDZ | 1.13 (0.32, 4.52) | 1.11 (0.30, 4.59) | 1.02 (0.26, 4.11) | 1.10 (0.31, 4.49) | 0.96 (0.27, 4.03) | 0.94 (0.25, 3.70) | 0.80 (0.21, 3.18) | 0.67 (0.18, 2.60) | 1.53 (0.39, 6.15) | 0.65 (0.18, 2.66) | 1.00 (0.45, 2.33) | 0.91 (0.23, 3.78) |
| 1.43 (0.21, 8.69) | 0.74 (0.12, 4.08) | 0.57 (0.14, 2.07) | 0.89 (0.22, 3.12) | UST | 0.98 (0.21, 4.50) | 0.90 (0.19, 4.07) | 0.98 (0.21, 4.54) | 0.86 (0.19, 4.09) | 0.82 (0.19, 3.69) | 0.71 (0.15, 3.24) | 0.59 (0.13, 2.61) | 1.36 (0.28, 5.91) | 0.58 (0.13, 2.58) | 0.88 (0.30, 2.51) | 0.81 (0.18, 3.66) |
| 1.44 (0.22, 9.83) | 0.76 (0.13, 4.18) | 0.58 (0.13, 2.05) | 0.90 (0.22, 3.35) | 1.02 (0.22, 4.85) | MIRI | 0.91 (0.19, 4.39) | 0.99 (0.22, 4.65) | 0.87 (0.19, 4.14) | 0.85 (0.18, 3.91) | 0.73 (0.15, 3.16) | 0.60 (0.13, 2.93) | 1.38 (0.30, 6.41) | 0.59 (0.13, 2.83) | 0.89 (0.30, 2.62) | 0.82 (0.17, 3.87) |
| 1.57 (0.23, 10.64) | 0.83 (0.14, 5.12) | 0.64 (0.14, 2.25) | 0.98 (0.24, 3.82) | 1.11 (0.25, 5.38) | 1.10 (0.23, 5.24) | GUSE | 1.09 (0.25, 5.27) | 0.95 (0.20, 4.54) | 0.92 (0.21, 4.24) | 0.79 (0.16, 3.89) | 0.65 (0.14, 3.22) | 1.51 (0.29, 7.41) | 0.64 (0.14, 3.11) | 0.97 (0.32, 2.94) | 0.90 (0.19, 4.23) |
| 1.45 (0.21, 9.24) | 0.76 (0.13, 4.23) | 0.58 (0.14, 2.05) | 0.91 (0.22, 3.26) | 1.02 (0.22, 4.86) | 1.01 (0.22, 4.59) | 0.92 (0.19, 4.06) | RISA | 0.87 (0.18, 4.15) | 0.84 (0.18, 3.74) | 0.73 (0.15, 3.49) | 0.60 (0.13, 2.76) | 1.38 (0.29, 6.26) | 0.59 (0.13, 2.81) | 0.90 (0.29, 2.63) | 0.83 (0.17, 3.81) |
| 1.66 (0.23, 10.78) | 0.87 (0.15, 4.75) | 0.67 (0.15, 2.39) | 1.04 (0.25, 3.77) | 1.17 (0.24, 5.40) | 1.15 (0.24, 5.23) | 1.05 (0.22, 4.96) | 1.15 (0.24, 5.43) | TOFA | 0.97 (0.20, 4.44) | 0.84 (0.17, 3.81) | 0.69 (0.15, 3.14) | 1.59 (0.32, 7.44) | 0.67 (0.14, 3.05) | 1.02 (0.34, 2.98) | 0.94 (0.19, 4.51) |
| 1.71 (0.25, 10.50) | 0.91 (0.15, 4.78) | 0.69 (0.16, 2.34) | 1.07 (0.27, 3.92) | 1.22 (0.27, 5.30) | 1.18 (0.26, 5.57) | 1.09 (0.24, 4.67) | 1.18 (0.27, 5.52) | 1.03 (0.23, 4.89) | UPA15 | 0.86 (0.18, 3.81) | 0.71 (0.16, 3.26) | 1.64 (0.32, 7.79) | 0.70 (0.15, 3.34) | 1.06 (0.36, 3.06) | 0.98 (0.32, 2.96) |
| 1.97 (0.29, 13.52) | 1.04 (0.17, 6.06) | 0.80 (0.19, 3.00) | 1.25 (0.31, 4.86) | 1.40 (0.31, 6.55) | 1.38 (0.32, 6.76) | 1.26 (0.26, 6.16) | 1.38 (0.29, 6.70) | 1.20 (0.26, 5.76) | 1.16 (0.26, 5.55) | FIL | 0.82 (0.19, 3.89) | 1.89 (0.39, 9.58) | 0.81 (0.17, 4.00) | 1.23 (0.42, 3.76) | 1.13 (0.24, 5.48) |
| 2.40 (0.36, 14.69) | 1.26 (0.23, 7.30) | 0.97 (0.23, 3.40) | 1.50 (0.38, 5.60) | 1.70 (0.38, 7.44) | 1.67 (0.34, 7.76) | 1.53 (0.31, 7.14) | 1.67 (0.36, 7.64) | 1.45 (0.32, 6.60) | 1.42 (0.31, 6.29) | 1.21 (0.26, 5.28) | OZAN | 2.32 (0.49, 10.56) | 0.99 (0.21, 4.43) | 1.49 (0.48, 4.27) | 1.38 (0.28, 6.41) |
| 1.05 (0.34, 3.11) | 0.55 (0.09, 3.21) | 0.42 (0.10, 1.63) | 0.65 (0.16, 2.59) | 0.73 (0.17, 3.60) | 0.72 (0.16, 3.32) | 0.66 (0.13, 3.40) | 0.73 (0.16, 3.48) | 0.63 (0.13, 3.08) | 0.61 (0.13, 3.13) | 0.53 (0.10, 2.55) | 0.43 (0.09, 2.06) | ETRO | 0.43 (0.09, 2.17) | 0.64 (0.22, 2.06) | 0.60 (0.12, 3.05) |
| 2.44 (0.36, 16.19) | 1.29 (0.21, 7.13) | 0.99 (0.23, 3.69) | 1.53 (0.38, 5.67) | 1.73 (0.39, 7.82) | 1.71 (0.35, 7.71) | 1.56 (0.32, 7.06) | 1.70 (0.36, 7.86) | 1.48 (0.33, 6.98) | 1.43 (0.30, 6.52) | 1.24 (0.25, 5.75) | 1.01 (0.23, 4.75) | 2.34 (0.46, 11.27) | ETRA | 1.52 (0.51, 4.51) | 1.40 (0.27, 6.39) |
| 1.62 (0.34, 7.43) | 0.85 (0.21, 3.42) | 0.65 (0.26, 1.37) | 1.00 (0.43, 2.22) | 1.14 (0.40, 3.38) | 1.12 (0.38, 3.31) | 1.03 (0.34, 3.15) | 1.11 (0.38, 3.45) | 0.98 (0.34, 2.95) | 0.94 (0.33, 2.80) | 0.81 (0.27, 2.36) | 0.67 (0.23, 2.06) | 1.55 (0.49, 4.51) | 0.66 (0.22, 1.97) | PBO | 0.92 (0.30, 2.88) |
| 1.74 (0.25, 11.68) | 0.92 (0.16, 5.16) | 0.70 (0.16, 2.61) | 1.10 (0.26, 4.26) | 1.23 (0.27, 5.59) | 1.21 (0.26, 5.83) | 1.11 (0.24, 5.32) | 1.20 (0.26, 5.91) | 1.07 (0.22, 5.19) | 1.02 (0.34, 3.10) | 0.88 (0.18, 4.14) | 0.72 (0.16, 3.56) | 1.67 (0.33, 8.46) | 0.71 (0.16, 3.65) | 1.08 (0.35, 3.37) | UPA30 |

**Supplementary Table S5** League table of any adverse events during the maintenance phase. Data are presented as odds ratios (ORs) with 95% confidence intervals (CIs). **Abbreviations:** ADA, adalimumab; ETRA, etrasimod; ETRO, etrolizumab; FIL, filgotinib; GOL, golimumab; GUSE, guselkumab; IFX, infliximab; MIRI, mirikizumab; OZAN, ozanimod; PBO, placebo; RISA, risankizumab; TOFA, tofacitinib; UPA15, upadacitinib 15 mg; UPA30, upadacitinib 30 mg; UST, ustekinumab; VDZ, vedolizumab

| IFX | ADA | GOL | VDZ | UST | MIRI | GUSE | RISA | TOFA | UPA | FIL | OZAN | ETRO | ETRA | PBO |
| --- | --- | --- | --- | --- | --- | --- | --- | --- | --- | --- | --- | --- | --- | --- |
| IFX | 0.97 (0.50, 2.06) | 0.41 (0.12, 1.33) | 0.54 (0.16, 1.62) | 1.56 (0.66, 4.35) | 1.34 (0.51, 3.62) | 1.76 (0.67, 5.04) | 3.20 (1.20, 8.46) | 1.10 (0.43, 2.65) | 1.20 (0.50, 3.00) | 0.71 (0.18, 2.47) | 0.42 (0.13, 1.28) | 0.56 (0.21, 1.59) | 0.35 (0.04, 2.10) | 0.74 (0.45, 1.24) |
| 1.03 (0.49, 1.99) | ADA | 0.43 (0.12, 1.31) | 0.56 (0.16, 1.67) | 1.60 (0.66, 4.35) | 1.39 (0.50, 3.62) | 1.81 (0.65, 5.18) | 3.29 (1.18, 8.57) | 1.12 (0.44, 2.66) | 1.24 (0.50, 3.04) | 0.73 (0.20, 2.40) | 0.44 (0.13, 1.25) | 0.59 (0.25, 1.35) | 0.36 (0.04, 2.17) | 0.76 (0.45, 1.21) |
| 2.41 (0.75, 8.62) | 2.34 (0.76, 8.30) | GOL | 1.32 (0.26, 5.83) | 3.78 (1.05, 15.70) | 3.24 (0.89, 13.89) | 4.28 (1.16, 18.77) | 7.75 (1.98, 33.32) | 2.62 (0.70, 10.84) | 2.91 (0.79, 11.46) | 1.68 (0.35, 8.51) | 1.01 (0.24, 4.67) | 1.38 (0.37, 5.75) | 0.85 (0.08, 6.69) | 1.77 (0.65, 5.78) |
| 1.86 (0.62, 6.32) | 1.79 (0.60, 6.45) | 0.76 (0.17, 3.81) | VDZ | 2.90 (0.88, 12.14) | 2.54 (0.66, 10.47) | 3.33 (0.87, 14.46) | 5.93 (1.54, 25.16) | 2.03 (0.57, 8.10) | 2.29 (0.63, 8.48) | 1.33 (0.26, 6.70) | 0.78 (0.17, 3.48) | 1.06 (0.28, 4.72) | 0.63 (0.07, 5.27) | 1.36 (0.50, 4.40) |
| 0.64 (0.23, 1.52) | 0.62 (0.23, 1.52) | 0.26 (0.06, 0.95) | 0.34 (0.08, 1.13) | UST | 0.85 (0.24, 2.58) | 1.13 (0.31, 3.66) | 2.04 (0.60, 6.17) | 0.70 (0.22, 2.00) | 0.77 (0.24, 2.24) | 0.45 (0.10, 1.71) | 0.27 (0.07, 0.89) | 0.36 (0.11, 1.13) | 0.22 (0.02, 1.46) | 0.47 (0.19, 0.99) |
| 0.74 (0.28, 1.95) | 0.72 (0.28, 2.01) | 0.31 (0.07, 1.12) | 0.39 (0.10, 1.52) | 1.17 (0.39, 4.13) | MIRI | 1.31 (0.39, 4.50) | 2.39 (0.70, 7.87) | 0.81 (0.25, 2.53) | 0.90 (0.28, 2.89) | 0.53 (0.12, 2.13) | 0.32 (0.08, 1.09) | 0.42 (0.12, 1.44) | 0.27 (0.03, 1.73) | 0.55 (0.23, 1.30) |
| 0.57 (0.20, 1.49) | 0.55 (0.19, 1.54) | 0.23 (0.05, 0.86) | 0.30 (0.07, 1.15) | 0.88 (0.27, 3.18) | 0.76 (0.22, 2.58) | GUSE | 1.80 (0.50, 6.27) | 0.62 (0.17, 1.95) | 0.67 (0.20, 2.21) | 0.40 (0.08, 1.61) | 0.24 (0.06, 0.90) | 0.32 (0.09, 1.12) | 0.20 (0.02, 1.31) | 0.42 (0.17, 1.01) |
| 0.31 (0.12, 0.83) | 0.30 (0.12, 0.84) | 0.13 (0.03, 0.50) | 0.17 (0.04, 0.65) | 0.49 (0.16, 1.68) | 0.42 (0.13, 1.43) | 0.56 (0.16, 2.01) | RISA | 0.34 (0.11, 1.06) | 0.38 (0.12, 1.21) | 0.22 (0.05, 0.92) | 0.13 (0.03, 0.47) | 0.18 (0.05, 0.63) | 0.11 (0.01, 0.74) | 0.23 (0.10, 0.54) |
| 0.91 (0.38, 2.34) | 0.89 (0.38, 2.28) | 0.38 (0.09, 1.44) | 0.49 (0.12, 1.75) | 1.42 (0.50, 4.64) | 1.23 (0.40, 3.98) | 1.61 (0.51, 5.81) | 2.92 (0.94, 9.29) | TOFA | 1.10 (0.39, 3.25) | 0.65 (0.15, 2.56) | 0.39 (0.10, 1.35) | 0.52 (0.16, 1.72) | 0.33 (0.03, 2.01) | 0.67 (0.33, 1.52) |
| 0.84 (0.33, 2.00) | 0.81 (0.33, 2.01) | 0.34 (0.09, 1.26) | 0.44 (0.12, 1.58) | 1.29 (0.45, 4.23) | 1.11 (0.35, 3.60) | 1.50 (0.45, 4.90) | 2.65 (0.83, 8.46) | 0.91 (0.31, 2.55) | UPA | 0.59 (0.13, 2.23) | 0.35 (0.09, 1.22) | 0.47 (0.16, 1.54) | 0.29 (0.03, 1.87) | 0.61 (0.28, 1.32) |
| 1.41 (0.40, 5.42) | 1.36 (0.42, 5.11) | 0.60 (0.12, 2.88) | 0.75 (0.15, 3.79) | 2.22 (0.58, 9.95) | 1.88 (0.47, 8.44) | 2.51 (0.62, 11.83) | 4.53 (1.09, 19.91) | 1.54 (0.39, 6.49) | 1.68 (0.45, 7.49) | FIL | 0.60 (0.13, 2.84) | 0.81 (0.19, 3.88) | 0.52 (0.05, 3.97) | 1.03 (0.34, 3.63) |
| 2.36 (0.78, 7.67) | 2.29 (0.80, 7.86) | 0.99 (0.21, 4.20) | 1.29 (0.29, 5.80) | 3.72 (1.13, 15.32) | 3.17 (0.92, 12.64) | 4.19 (1.11, 17.95) | 7.64 (2.15, 29.63) | 2.57 (0.74, 9.77) | 2.86 (0.82, 10.66) | 1.68 (0.35, 7.52) | OZAN | 1.34 (0.38, 5.70) | 0.84 (0.09, 6.65) | 1.73 (0.67, 5.23) |
| 1.78 (0.63, 4.66) | 1.70 (0.74, 4.00) | 0.72 (0.17, 2.71) | 0.94 (0.21, 3.54) | 2.75 (0.88, 9.28) | 2.36 (0.69, 8.20) | 3.11 (0.89, 11.10) | 5.65 (1.59, 18.63) | 1.91 (0.58, 6.10) | 2.11 (0.65, 6.41) | 1.24 (0.26, 5.14) | 0.75 (0.18, 2.66) | ETRO | 0.63 (0.06, 4.35) | 1.29 (0.54, 2.99) |
| 2.84 (0.48, 23.77) | 2.74 (0.46, 23.16) | 1.18 (0.15, 11.87) | 1.58 (0.19, 14.22) | 4.48 (0.69, 42.10) | 3.76 (0.58, 35.71) | 4.98 (0.77, 48.44) | 9.01 (1.34, 87.35) | 3.03 (0.50, 29.65) | 3.39 (0.53, 32.68) | 1.93 (0.25, 22.01) | 1.19 (0.15, 11.55) | 1.58 (0.23, 15.44) | ETRA | 2.06 (0.38, 16.66) |
| 1.36 (0.81, 2.21) | 1.32 (0.82, 2.20) | 0.56 (0.17, 1.55) | 0.74 (0.23, 2.01) | 2.12 (1.01, 5.13) | 1.83 (0.77, 4.30) | 2.39 (0.99, 5.96) | 4.36 (1.86, 10.21) | 1.49 (0.66, 3.07) | 1.63 (0.76, 3.52) | 0.97 (0.28, 2.94) | 0.58 (0.19, 1.49) | 0.77 (0.33, 1.84) | 0.49 (0.06, 2.60) | PBO |

**Supplementary Table S6** League table of serious adverse events during the induction phase. Data are presented as odds ratios (ORs) with 95% confidence intervals (CIs). Abbreviations: ADA, adalimumab; ETRA, etrasimod; ETRO, etrolizumab; FIL, filgotinib; GOL, golimumab; GUSE, guselkumab; IFX, infliximab; MIRI, mirikizumab; OZAN, ozanimod; PBO, placebo; RISA, risankizumab; TOFA, tofacitinib; UPA15, upadacitinib 15 mg; UPA30, upadacitinib 30 mg; UST, ustekinumab; VDZ, vedolizumab. NC,Not calculable due to sparse data or zero events in the corresponding treatment arm, resulting in unstable wide credible intervals.

| IFX | ADA | GOL | VDZ | UST | MIRI | GUSE | RISA | TOFA | UPA15 | FIL | OZAN | ETRO | ETRA | PBO | UPA30 |
| --- | --- | --- | --- | --- | --- | --- | --- | --- | --- | --- | --- | --- | --- | --- | --- |
| IFX | 0.59 (0.02, 23.16) | 0.59 (0.04, 18.19) | 0.77 (0.04, 15.83) | 1.10 (0.04, 29.87) | 1.81 (0.05, 60.47) | 1.60 (0.05, 48.96) | 1.22 (0.04, 38.50) | 0.95 (0.03, 25.91) | 1.50 (0.05, 41.88) | NC | 1.15 (0.04, 40.90) | 0.62 (0.09, 3.92) | 0.69 (0.02, 24.05) | 0.81 (0.05, 13.36) | 1.94 (0.07, 53.62) |
| 1.69 (0.04, 64.39) | ADA | 0.97 (0.10, 21.00) | 1.29 (0.20, 8.84) | 1.88 (0.11, 33.71) | 3.06 (0.17, 60.48) | 2.76 (0.18, 46.41) | 2.05 (0.12, 42.43) | 1.57 (0.10, 33.42) | 2.53 (0.15, 46.68) | NC | 1.98 (0.13, 44.81) | 1.06 (0.05, 21.36) | 1.17 (0.07, 24.84) | 1.36 (0.17, 13.87) | 3.22 (0.20, 64.53) |
| 1.69 (0.05, 28.00) | 1.03 (0.05, 9.71) | GOL | 1.29 (0.14, 6.12) | 1.91 (0.13, 14.91) | 3.09 (0.20, 26.24) | 2.72 (0.18, 24.37) | 2.06 (0.15, 19.23) | 1.61 (0.11, 14.59) | 2.47 (0.18, 20.46) | NC | 1.95 (0.14, 16.81) | 1.04 (0.06, 9.11) | 1.18 (0.08, 9.98) | 1.37 (0.25, 4.67) | 3.25 (0.23, 26.85) |
| 1.31 (0.06, 27.43) | 0.77 (0.11, 4.95) | 0.78 (0.16, 7.23) | VDZ | 1.46 (0.17, 14.19) | 2.39 (0.25, 22.84) | 2.14 (0.25, 20.75) | 1.58 (0.19, 16.74) | 1.25 (0.14, 12.15) | 1.96 (0.21, 17.33) | NC | 1.52 (0.17, 16.17) | 0.81 (0.08, 8.63) | 0.90 (0.10, 10.24) | 1.06 (0.37, 3.64) | 2.52 (0.30, 24.36) |
| 0.91 (0.03, 26.17) | 0.53 (0.03, 9.44) | 0.52 (0.07, 7.61) | 0.69 (0.07, 5.92) | UST | 1.63 (0.11, 23.63) | 1.45 (0.11, 20.85) | 1.09 (0.08, 18.83) | 0.85 (0.06, 12.53) | 1.35 (0.09, 17.33) | NC | 1.04 (0.07, 15.17) | 0.56 (0.03, 8.43) | 0.63 (0.05, 9.19) | 0.73 (0.11, 4.79) | 1.73 (0.13, 24.60) |
| 0.55 (0.02, 18.79) | 0.33 (0.02, 5.99) | 0.32 (0.04, 4.97) | 0.42 (0.04, 4.03) | 0.61 (0.04, 8.96) | MIRI | 0.89 (0.06, 14.53) | 0.66 (0.05, 10.14) | 0.52 (0.04, 8.47) | 0.81 (0.05, 12.38) | NC | 0.65 (0.04, 10.56) | 0.34 (0.02, 5.89) | 0.38 (0.03, 6.34) | 0.44 (0.07, 3.32) | 1.07 (0.08, 17.20) |
| 0.62 (0.02, 19.57) | 0.36 (0.02, 5.56) | 0.37 (0.04, 5.54) | 0.47 (0.05, 3.99) | 0.69 (0.05, 9.44) | 1.12 (0.07, 15.95) | GUSE | 0.75 (0.05, 11.50) | 0.58 (0.04, 9.11) | 0.91 (0.06, 12.17) | NC | 0.72 (0.04, 10.28) | 0.38 (0.02, 5.72) | 0.43 (0.03, 6.68) | 0.50 (0.07, 3.46) | 1.19 (0.08, 17.71) |
| 0.82 (0.03, 24.98) | 0.49 (0.02, 8.36) | 0.48 (0.05, 6.74) | 0.63 (0.06, 5.27) | 0.92 (0.05, 12.84) | 1.51 (0.10, 21.41) | 1.34 (0.09, 19.48) | RISA | 0.80 (0.05, 11.62) | 1.23 (0.07, 17.04) | NC | 0.96 (0.06, 14.30) | 0.52 (0.03, 7.48) | 0.59 (0.03, 9.04) | 0.67 (0.09, 4.34) | 1.58 (0.10, 24.19) |
| 1.06 (0.04, 29.27) | 0.63 (0.03, 10.09) | 0.62 (0.07, 8.93) | 0.80 (0.08, 6.98) | 1.17 (0.08, 17.52) | 1.92 (0.12, 27.57) | 1.71 (0.11, 26.04) | 1.25 (0.09, 21.26) | TOFA | 1.55 (0.10, 22.44) | NC | 1.23 (0.08, 18.71) | 0.65 (0.04, 9.67) | 0.73 (0.05, 12.52) | 0.86 (0.13, 5.50) | 2.01 (0.15, 33.79) |
| 0.67 (0.02, 18.44) | 0.40 (0.02, 6.55) | 0.40 (0.05, 5.63) | 0.51 (0.06, 4.68) | 0.74 (0.06, 11.64) | 1.24 (0.08, 18.20) | 1.09 (0.08, 16.13) | 0.81 (0.06, 13.46) | 0.65 (0.04, 9.62) | UPA15 | NC | 0.78 (0.05, 12.17) | 0.41 (0.03, 6.94) | 0.46 (0.03, 7.48) | 0.54 (0.08, 3.80) | 1.30 (0.19, 8.81) |
| NC | NC | NC | NC | NC | NC | NC | NC | NC | NC | FIL | NC | NC | NC | NC | NC |
| 0.87 (0.02, 24.00) | 0.51 (0.02, 7.92) | 0.51 (0.06, 7.01) | 0.66 (0.06, 5.80) | 0.96 (0.07, 13.66) | 1.55 (0.09, 23.40) | 1.40 (0.10, 22.32) | 1.04 (0.07, 16.53) | 0.81 (0.05, 11.86) | 1.29 (0.08, 20.55) | NC | OZAN | 0.54 (0.03, 8.19) | 0.59 (0.04, 10.56) | 0.70 (0.10, 5.18) | 1.67 (0.11, 26.11) |
| 1.61 (0.25, 10.86) | 0.94 (0.05, 20.13) | 0.96 (0.11, 15.55) | 1.23 (0.12, 11.91) | 1.78 (0.12, 28.93) | 2.95 (0.17, 48.57) | 2.62 (0.17, 40.93) | 1.94 (0.13, 34.88) | 1.54 (0.10, 24.39) | 2.42 (0.14, 36.63) | NC | 1.85 (0.12, 33.51) | ETRO | 1.11 (0.07, 21.30) | 1.31 (0.18, 10.00) | 3.13 (0.21, 49.64) |
| 1.45 (0.04, 41.26) | 0.86 (0.04, 13.83) | 0.85 (0.10, 12.78) | 1.11 (0.10, 9.66) | 1.60 (0.11, 20.25) | 2.60 (0.16, 37.88) | 2.34 (0.15, 34.24) | 1.71 (0.11, 29.74) | 1.37 (0.08, 20.50) | 2.16 (0.13, 29.32) | NC | 1.69 (0.09, 28.22) | 0.90 (0.05, 13.47) | ETRA | 1.17 (0.17, 8.28) | 2.80 (0.18, 40.15) |
| 1.23 (0.07, 20.58) | 0.73 (0.07, 5.82) | 0.73 (0.21, 3.94) | 0.95 (0.27, 2.71) | 1.36 (0.21, 9.34) | 2.25 (0.30, 14.81) | 1.99 (0.29, 13.89) | 1.49 (0.23, 11.28) | 1.17 (0.18, 7.55) | 1.85 (0.26, 12.44) | NC | 1.43 (0.19, 10.01) | 0.76 (0.10, 5.51) | 0.86 (0.12, 5.99) | PBO | 2.37 (0.36, 15.60) |
| 0.52 (0.02, 13.83) | 0.31 (0.02, 4.92) | 0.31 (0.04, 4.35) | 0.40 (0.04, 3.34) | 0.58 (0.04, 7.77) | 0.94 (0.06, 13.01) | 0.84 (0.06, 12.38) | 0.63 (0.04, 10.50) | 0.50 (0.03, 6.83) | 0.77 (0.11, 5.16) | NC | 0.60 (0.04, 9.04) | 0.32 (0.02, 4.75) | 0.36 (0.02, 5.49) | 0.42 (0.06, 2.76) | UPA30 |

**Supplementary Table S7** League table of serious adverse events during the maintenance phase. Data are presented as odds ratios (ORs) with 95% confidence intervals (CIs). Abbreviations: ADA, adalimumab; ETRA, etrasimod; ETRO, etrolizumab; FIL, filgotinib; GOL, golimumab; GUSE, guselkumab; IFX, infliximab; MIRI, mirikizumab; OZAN, ozanimod; PBO, placebo; RISA, risankizumab; TOFA, tofacitinib; UPA15, upadacitinib 15 mg; UPA30, upadacitinib 30 mg; UST, ustekinumab; VDZ, vedolizumab. NC,Not calculable due to sparse data or zero events in the corresponding treatment arm, resulting in unstable wide credible intervals

| Treatment | SUCRA (%) - Primary Analysis | Rank - Primary Analysis | SUCRA (%) - Sensitivity Analysis | Rank - Sensitivity Analysis |
| --- | --- | --- | --- | --- |
| Upadacitinib | 99.9 | 1 | 99.9 | 1 |
| Risankizumab | 78.33 | 2 | 75.15 | 2 |
| Ustekinumab | 71.94 | 3 | 72.29 | 3 |
| Infliximab | 71.26 | 4 | 71.47 | 4 |
| Ozanimod | 70.34 | 5 | 68.93 | 5 |
| Tofacitinib | 63.46 | 8 | 68.78 | 6 |
| Guselkumab | 67.9 | 6 | 65.52 | 7 |
| Golimumab | 64.7 | 7 | 63.75 | 8 |
| Etrasimod | 52.62 | 9 | 51.67 | 9 |
| Vedolizumab | 46.05 | 10 | 48.13 | 10 |
| Mirikizumab | 33.56 | 11 | 30.45 | 11 |
| Adalimumab | 28.11 | 13 | 29.14 | 12 |
| Filgotinib | 28.87 | 12 | 28.98 | 13 |
| Etrolizumab | 19.99 | 14 | 23.03 | 14 |
| Placebo | 4.05 | 15 | 4.12 | 15 |

**Supplementary Table S8** Cumulative ranking probabilities (SUCRA) and treatment hierarchies for induction clinical remission: primary analysis versus pooled sensitivity analysis. Abbreviations: SUCRA, Surface Under the Cumulative Ranking curve. This table presents the results of the targeted sensitivity analysis, in which the HIBISCUS I and II trials were integrated into a single pooled node utilizing their original, un-imputed combined adalimumab data. The consistent top-ranking positions of Upadacitinib and Risankizumab confirm that the data imputation approach utilized in the primary analysis did not compromise or alter the overall efficacy hierarchy

| Treatment | Induction Clinical Remission (%) | Induction Endoscopic Improvement (%) | Induction DAEs (%) | Maintenance Clinical Remission (%) | Maintenance Endoscopic Improvement (%) | Maintenance DAEs (%) |
| --- | --- | --- | --- | --- | --- | --- |
| Adalimumab | 28.1 | 16.2 | 29.7 | 45.8 | 29.3 | 32.2 |
| Etrasimod | 52.6 | 61.1 | 9.7 | 89.1 | 51.7 | 34.4 |
| Etrolizumab | 20 | 47.3 | 25.3 | 20.9 | 32.5 | 55.4 |
| Filgotinib | 28.9 | 50 | 36.3 | 76.3 | 67.9 | 21.3 |
| Golimumab | 64.7 | 31.6 | 69.1 | 62.4 | 41.7 | 19.4 |
| Guselkumab | 67.9 | 36.6 | 57.3 | 63.2 | 95 | 64.1 |
| Infliximab | 71.3 | 68.6 | 51.5 | 37.5 | 56 | 59.9 |
| Mirikizumab | 33.6 | 46.5 | 70.8 | 45.2 | 64.2 | 87.9 |
| Ozanimod | 70.3 | 73 | 35 | 41.2 | 31.4 | 60.8 |
| Risankizumab | 78.3 | 92.6 | 76.1 | 25.7 | 27.3 | 36 |
| Tofacitinib | 63.5 | 66.9 | 36.6 | 77.7 | 75.8 | 53.1 |
| Upadacitinib | 99.9 | 99.9 | 69.5 | - | - | - |
| Upadacitinib 15 mg | - | - | - | 79.2 | 79.5 | 70.4 |
| Upadacitinib 30 mg | - | - | - | 92.6 | 94.9 | 50.3 |
| Ustekinumab | 71.9 | 78.9 | 98.9 | 31.4 | 44.4 | 80.4 |
| Vedolizumab | 46 | 27.3 | 54.5 | 60.5 | 57.7 | 55.8 |
| Placebo | 4 | 3.8 | 29.9 | 4.6 | 4.1 | 18.5 |

**Supplementary Table S9** Surface Under the Cumulative Ranking curve (SUCRA) probabilities for all advanced therapies across primary efficacy and tolerability outcomes. Abbreviations: SUCRA, Surface Under the Cumulative Ranking curve; DAE, discontinuation due to adverse events. SUCRA values are expressed as percentages (%). A higher SUCRA value indicates a higher probability that the treatment is among the top-ranking interventions for efficacy. Conversely, for the tolerability endpoint (DAE), a higher SUCRA value corresponds to a higher probability of safety (i.e., a lower risk of discontinuation). Values must be interpreted in conjunction with their corresponding odds ratios (ORs) and 95% credible intervals, as marginal numerical differences in SUCRA do not necessarily imply statistically significant clinical differences.

**Supplementary Figure S1** Efficacy of targeted therapies in the biologic-naïve cohort. Forest plots [or League tables, demonstrating the network meta-analysis results for (A) clinical remission during the induction phase, (B) endoscopic improvement during the induction phase, (C)clinical remission during the maintenance phase, and (D) endoscopic improvement during the maintenance phase. All effect sizes are presented as Odds Ratios (ORs) compared with placebo. OR = Odds Ratio; CrI = Credible Interval. Bold text/lines indicate statistically significant differences compared with placebo (i.e., the 95% CrI does not include 1). In the maintenance phase (C and D), wider credible intervals reflect greater statistical uncertainty due to limited data; notably, no active drug achieved statistical significance for endoscopic improvement in this specific phase.

**Supplementary Figure S2** Efficacy of targeted therapies in the biologic-exposed (refractory) cohort. Forest plots demonstrating the network meta-analysis results for (A) clinical remission during the induction phase, (B) endoscopic improvement during the induction phase, (C) clinical remission during the maintenance phase, and (D) endoscopic improvement during the maintenance phase. All effect sizes are presented as Odds Ratios (ORs) compared with placebo. OR = Odds Ratio; CrI = Credible Interval. Bold text/lines indicate statistically significant differences compared with placebo (i.e., the 95% CrI does not include 1). Upadacitinib 30 mg and Guselkumab emerged as the leading therapies across phases in this refractory population. Conversely, certain agents, such as Filgotinib (induction) and Etrolizumab (maintenance), did not reach statistical significance for clinical remission compared with placebo.

**Supplementary Figure S3** Comparison-adjusted funnel plots assessing publication bias across primary efficacy outcomes. (A) Induction phase: clinical remission. (B) Induction phase: endoscopic improvement.  (C)Maintenance phase: endoscopic improvement. (D) Maintenance phase: clinical remission (original analysis). The symmetrical distribution of effect estimates in panels A, B, and C indicates the absence of significant publication bias for these outcomes
